# Resveratrol: Sensitising CD44+ Cervical Cancer Cells to Carboplatin and Mitigating Metastasis

**DOI:** 10.1002/cam4.71194

**Published:** 2025-09-01

**Authors:** Cayleigh de Sousa, Carla Eksteen, Anna‐Mart Engelbrecht

**Affiliations:** ^1^ Department of Physiological Sciences, Faculty of Science Stellenbosch University Stellenbosch South Africa

**Keywords:** carboplatin, CD44, cervical cancer, chemo‐sensitivity, metastasis

## Abstract

**Background:**

Cervical cancer remains a leading cause of cancer‐related mortality among women globally. Persistent infection with high‐risk human papillomavirus drives cervical carcinogenesis, and treatment outcomes are frequently challenged by metastasis and chemoresistance. The transmembrane glycoprotein cluster of differentiation 44 (CD44), a marker associated with cancer stem cells (CSCs), has emerged as a critical mediator of both processes in cervical cancer.

**Objective:**

This review aims to critically evaluate current evidence on the role of CD44 in cervical cancer progression, metastasis, and treatment resistance. It also explores the potential of resveratrol, a naturally occurring polyphenol with known anticancer properties, as a chemo‐sensitizing agent to carboplatin therapy.

**Methods:**

A comprehensive review of the literature was conducted using databases such as PubMed, Google Scholar, and Scopus to identify studies that investigate CD44‐mediated mechanisms in cervical cancer, as well as the modulatory and mechanistic effects of resveratrol on CD44 and chemoresistance across various cancer types.

**Results:**

CD44 has been consistently implicated in promoting drug resistance, epithelial‐to‐mesenchymal transition (EMT), and stemness in cervical cancer. Resveratrol has demonstrated antimetastatic and chemo‐sensitizing effects in several malignancies, such as colorectal and breast cancers, often through modulation of CD44 and associated pathways. However, direct evidence in cervical cancer remains limited.

**Conclusion:**

Current findings suggest a promising therapeutic avenue for combining resveratrol with carboplatin to overcome CD44‐mediated treatment resistance and metastasis in cervical cancer. Nonetheless, further cervical‐specific studies are needed to validate this approach. A clearer understanding of this relationship may facilitate lower chemotherapy dosing, reduced toxicity, and improved clinical outcomes.

## Graphical abstract

Resveratrol reduces metastasis and EMT by inhibiting aberrant signaling pathways, promoting E‐cadherin transcription, and reducing mesenchymal marker expression (vimentin, N‐cadherin, and slug). In addition, it inhibits E6/E7 oncoproteins and reduces CSC/CD44 levels in the tumor microenvironment, enhancing carboplatin sensitivity and reducing drug resistance in cervical cancer.

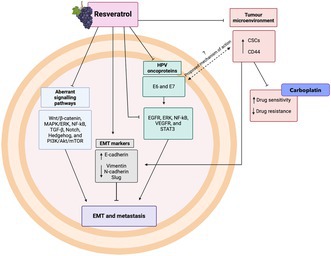

## 1 Introduction

Cervical cancer continues to be a serious public health threat among women worldwide [1], with high‐risk human papillomavirus (HPV) infections being central to cervical malignancy. Recurring high‐risk HPV infections account for 90% of all diagnosed cases [2], and although persisting HPV infections are necessary for cervical tumourigenesis, they are insufficient when acting alone. It is the combination of recurring high‐risk HPV subtype infections, particularly HPV‐16 and HPV‐18, alongside other established cancer‐associated risk factors that prompt cervical malignant behaviour [3]. HPV's tumourigenesis potential is primarily attributed to its high‐risk subtype oncogene production of *E6* and *E7*, which promote dysregulated cell signalling pathway activities, immune evasion, and genetic instability [4]. Therefore, early detection and preventative strategies, such as access to frequent HPV screening appointments and vaccination programs, could prove highly beneficial in preventing cervical cancer [1]. However, due to apparent global socioeconomic disparities, low‐to‐middle‐income countries (LMICs) have less access to screening and vaccination resources, thus placing these countries at a greater predisposition for cervical disease. Currently, 85% of all global cervical cancer‐related deaths occur in LMICs, with the continent of Africa possessing the highest age‐standardised incidence mortality rates [4].

Appropriate cervical cancer treatment strategies are largely dependent on the staging system of the International Federation of Gynaecology and Obstetrics (FIGO) [5]. Generally, early‐stage disease treatment approaches include surgical excision. For recurrent/advanced cases, often presenting with greater treatment challenges, concurrent chemoradiation techniques are favored as chemotherapeutic drugs sensitize cervical cancer cells to the radiation therapy [6, 7]. Specifically, the use of platinum‐based chemotherapeutic drugs, such as cisplatin and carboplatin, is commonly prescribed for the treatment of cervical disease [8]. Carboplatin exists as a derivative of cisplatin, differing in its structure, and is known to have a lowered degree of toxicity [9], making it an attractive treatment option for advanced‐stage cervical cancer patients.

Despite these treatment approaches, metastatic and recurrent cervical malignancies continue to persist [5]. The accelerated proliferation of tumour cells frequently leads to the emergence of genetic mutations that confer treatment resistance, ultimately facilitating metastasis [10, 11]. This metastatic process is characterised by the development of secondary tumours at distant sites away from the primary/original tumour [12]. To improve patient survival outcomes, identifying and understanding the role various biomarkers play within the tumourigenesis process is vital for oncological treatment advancement strategies. One such biomarker includes the metastatic and treatment resistance‐promoting stem cell marker, cluster of differentiation 44 (CD44). CD44 is highly expressed on the surface of cervical cancer‐associated cells within the tumour microenvironment (TME), including immune cells [13], cancer‐associated fibroblasts (CAFs), and cancer stem cells (CSCs). This heightened cellular expression contributes to treatment challenges for these patients [14].

Furthermore, investigations are being conducted into the use of naturally occurring treatment options, such as the phytochemical resveratrol, which possesses anticancer potential and lower toxicity to healthy cells. These alternatives are being sought out and introduced into the field of oncology in hopes of avoiding or reducing apparent treatment side effects. Thus, this review aims to evaluate whether resveratrol treatment could potentially enhance the chemo‐sensitivity of cervical cancer cells to the chemotherapeutic drug, carboplatin, by mitigating the pathological effects of CD44‐induced metastasis and chemoresistance within cervical cancer patients.

## 2 Methods

To identify relevant literature on resveratrol's role in cervical cancer, particularly its interaction with CD44 and potential to enhance carboplatin sensitivity, a structured literature search was conducted using Google Scholar, PubMed, and Scopus. The following key terms were used: “cervical cancer” AND “metastasis” “cervical cancer” OR “treatment resistance” OR “chemoresistance” “cervical cancer” AND “resveratrol” “cervical cancer” AND “CD44” “cervical cancer” AND “resveratrol” AND “CD44” “cervical cancer” AND “carboplatin” “carboplatin” OR “platinum drugs” “carboplatin” AND “resveratrol,” “carboplatin” AND “sensitisation” “cervical cancer” AND “carboplatin” AND “chemo‐sensitisation”.

Studies focusing specifically on cervical cancer models, CD44 expression, resveratrol's mechanisms, and carboplatin interactions were prioritised. Key findings were extracted and thematically synthesised to critically evaluate the current evidence and identify knowledge gaps.

Figures were designed using BioRender software.

## 3 The Role of CD44 in Cervical Cancer Tumor Progression

The metastatic and treatment‐resistant potential of cervical cancer cells constitutes a significant aspect of cervical cancer tumor progression. In addition, CD44's abnormal expression on the surface of tumor‐associated cells facilitates the upregulation of metastasis, survival, and stemness‐promoting pathways, ultimately resulting in treatment challenges [15]. Clinical evidence supporting CD44's pathogenic role in cervical cancer is summarized in Table 1.

**TABLE 1:** A summarized representation of studies encompassing CD44's pathogenic role in cervical cancer.

Trial/study	Stage	Number of patients	Treatment	Outcome	References
Corre‐lational study	Various	26	None (Visualised marker expression levels using immuno‐histochemistry)	Elevated CD44 levels correlated with increasing degrees of cervical malignancy	[16]
Meta‐analysis (22 studies)	N/A	N/A	Various	High CD44 CCSC marker expression levels = poor disease‐free and overall survival	[17]
Prospective study	IB—IVA	150	Weekly cisplatin, concurrent radiation, and brachytherapy	Increased CD44 = poor disease locoregional control	[18]
Phase II	IIB—IVA	126	Cisplatin radio‐chemotherapy or (vinorelbine, docetaxel, ifosfamide‐vinorelbine‐cisplatin)	Tumour's exhibiting expansive growth were CD44 positive	[19]
Cross‐sectional study	Various	100	None (serum analysis)	Significant increases in CD44 levels in cervical cancer patents vs. healthy patients	[20]
Retro‐spective study	IB—IIA	21	Preoperative epirubicin, cisplatin, bleomycin, and etoposide	CD44v6 = prognostic marker indicating the response to neoadjuvant chemotherapy	[21]

Abbreviations: CCSC, Cervical cancer stem cell; CD44, Cluster of differentiation 44; CD44v6, Cluster of differentiation 44 variant 6; N/A, Not applicable.

While CD44 is naturally expressed at homeostatic levels throughout the body, facilitating cell–cell integrity and cell‐extracellular matrix (ECM) communication, its expression is significantly elevated within the TME, including the cervical cancer TME [15, 22, 23].

### 3.1 CD44 as a Cancer Stem Cell (CSC) Marker

CD44 is commonly expressed on the surface of CSCs, which are abundant within the TME and facilitate enhanced stemness and treatment resistance potential [24, 25]. Not only are CSCs recognized as a potential prognostic marker for cervical cancer patients [18], but they also facilitate CD44's communicative crosstalk between the ECM and the CSC surface. This communication induces metastatic signaling via CD44's ligand preference for the ECM component, hyaluronic acid (HA).

Hyaluronic acid is commonly expressed at high levels within the TME. Therefore, increased HA‐CD44 binding induces aberrant CD44 target gene transcription [14], which ultimately promotes enhanced cell survival, cell invasion, and metastatic cell signaling pathways, including CD44 self‐transcription [22]. Furthermore, CD44‐HA binding has a roll‐on effect that enhances the treatment resistance properties of cancer cells via increased *multidrug resistance protein 1* (*MDR1*) expression and epithelial‐to‐mesenchymal transition (EMT) pathway upregulation [26]. Normally, *MDR1* expression promotes cytotoxic cellular protection. However, abnormally enhanced *MDR1* expression levels, observed in CD44‐positive cervical cancer cells, exacerbate this resistant phenomenon and favor the formation of treatment‐resistant cancer cells, including CSCs [25]. Furthermore, aberrant cervical cancer stem cell (CCSC) CD44 overexpression is frequently linked to metastasis in cervical cancer patients. CD44 can be utilized as both a chemoresistant and prognostic marker with the added effect of high‐risk HPV oncogenes, *E6* and *E7*, enhancing their self‐renewal capacity [27]. For example, a systematic review performed by Fahmi et al. (2021) identified that increased CD44 expression levels in cervical cancer cells were commonly associated with a more aggressive tumor phenotype, as well as highlighting a correlation between elevated CD44 levels and reduced overall survival in patients with cervical cancer [17]. In addition to CSCs, the stromal cell subpopulation of CAFs found within the TME promotes the treatment resistance phenotype of CSCs. Similarly, circulating tumor cells (CTCs), commonly observed in the blood/lymph systems of metastatic cancer patients, express high levels of CD44 on their surface, ultimately underscoring the enhanced tumorigenesis, metastasis, treatment resistance, and immune evasion properties of tumor‐associated CD44‐positive cells [28, 29]. Moreover, CD44's well‐known three‐domain structure facilitates the formation of structural CD44 isoforms, which favor metastatic effects as observed in cervical cancer cells.

### 3.2 Cervical Tumour‐Promoting CD44 Variants

**FIGURE 1:**
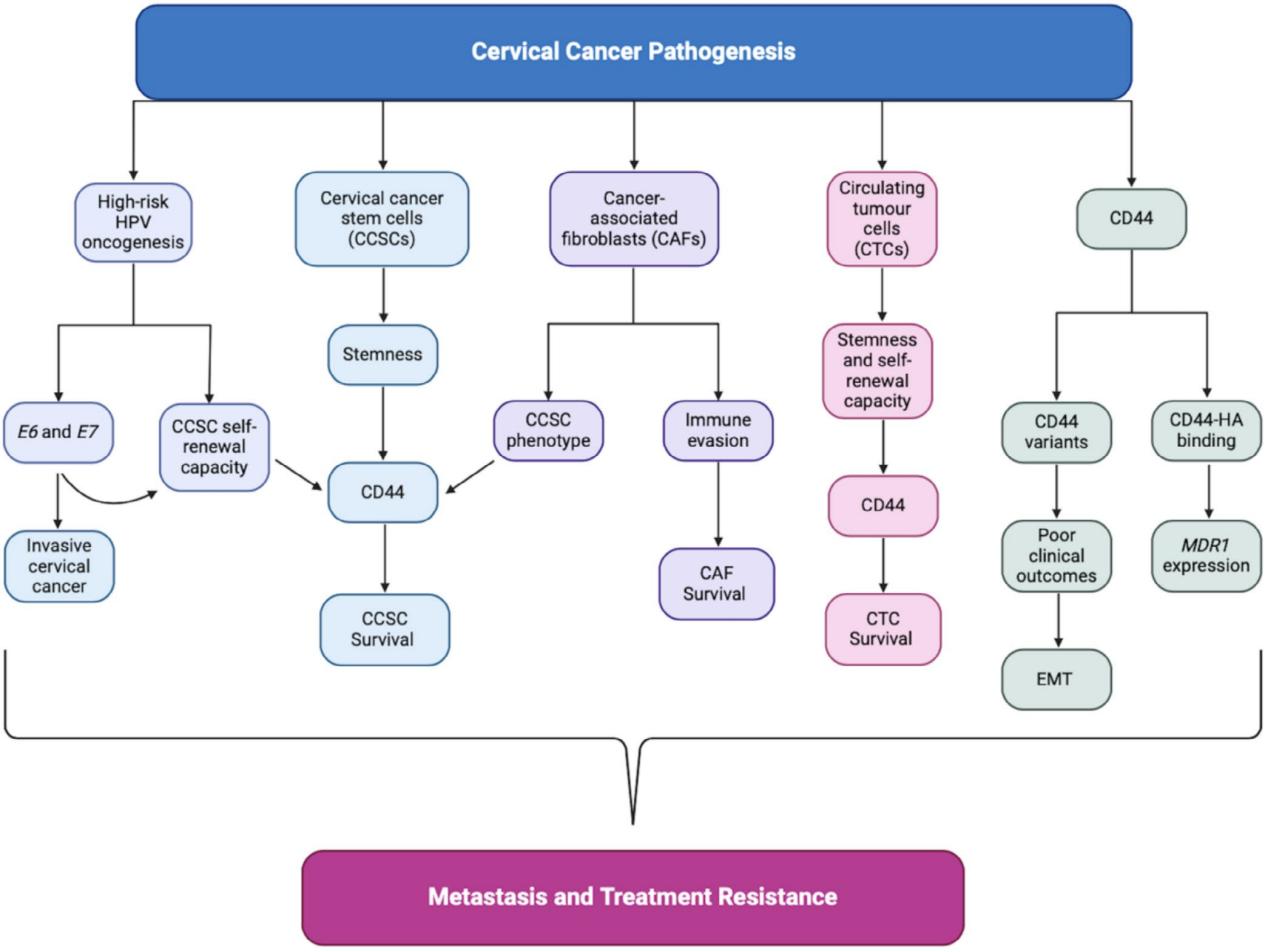
Summary of cervical cancer contributing pathogenic factors. High‐risk HPV oncogenesis includes the production of oncogenes, *E6* and *E7*, which promote CCSC production, self‐renewal activities, enhanced CD44 expression, as well as CIN conversion‐induced invasive cervical cancer. Similarly, CCSCs favor increased CD44‐induced survival. CAFs are responsible for upregulating both immune evasion and survival‐promoting properties. Furthermore, CTCs enhance stemness and CD44 expression in cervical cancer cells. Lastly, CD44's variant isoforms and HA ligand binding preference promote poor clinical outcomes due to enhanced EMT pathway upregulation and increased *MDR1* expression levels, respectively. Ultimately, all factors contribute to the metastatic and treatment resistance potential of cervical cancer cells. CAFs, Cancer‐associated fibroblasts; CCSC, Cervical cancer stem cell; CD44, Cluster of differentiation 44; CIN, Cervical intraepithelial neoplasia; CTCs, Circulating tumor cells; EMT, Epithelial‐to‐mesenchymal transition; HA, Hyaluronic acid; HPV, Human papillomavirus; MDR1, Multidrug resistance 1. Created with BioRender.com.

Various CD44 variants (CD44v), derived from ribonucleic acid (RNA) splicing of the standard CD44 isoform (CD44s), exist in different combinations and expression levels across various types of cancers [15]. The presence of CD44 variants has been associated with poor clinical outcomes and enhanced EMT signaling pathway mechanisms [29]. For example, CD44v6 is known to promote cytoskeleton changes and tumor cell proliferation via mitogen‐activated protein kinase (MAPK)/Ras and vascular endothelial growth factor (VEGF) signaling pathways upregulation [26, 29]. Literature suggests the CD44v6–8 isoforms are commonly associated with metastatic‐promoting cervical cancer pathogenic effects [15, 30], with CD44v6 specifically observed to promote EMT and invasion [31]. Taken together, all CD44's metastatic‐promoting factors ultimately enhance treatment resistance pathways involved in cervical tumorigenesis (summarised in Figure 1).

Despite the above CD44‐induced treatment challenges, various chemotherapeutic agents remain effective in the treatment of cervical malignancy.

## 4 Carboplatin in Cervical Cancer Treatment

The use of platinum‐based chemotherapeutic drugs is an attractive therapeutic avenue in treating cervical cancer [8]. Interestingly, advanced‐stage cervical cancer patients in LMICs, such as in Sub‐Saharan Africa, commonly receive a cisplatin‐based chemotherapy treatment strategy [32]. However, the observed nephrotoxic and neurotoxic side effects associated with the frequently prescribed cervical cancer drug prompted the pursuit of a less toxic but equally effective alternative, such as carboplatin [33]. Interestingly, despite their distinct chemical differences and toxicity profiles, both utilize analogous mechanisms of action [33].

### 4.1 Carboplatin's Mechanism of Action

**FIGURE 2:**
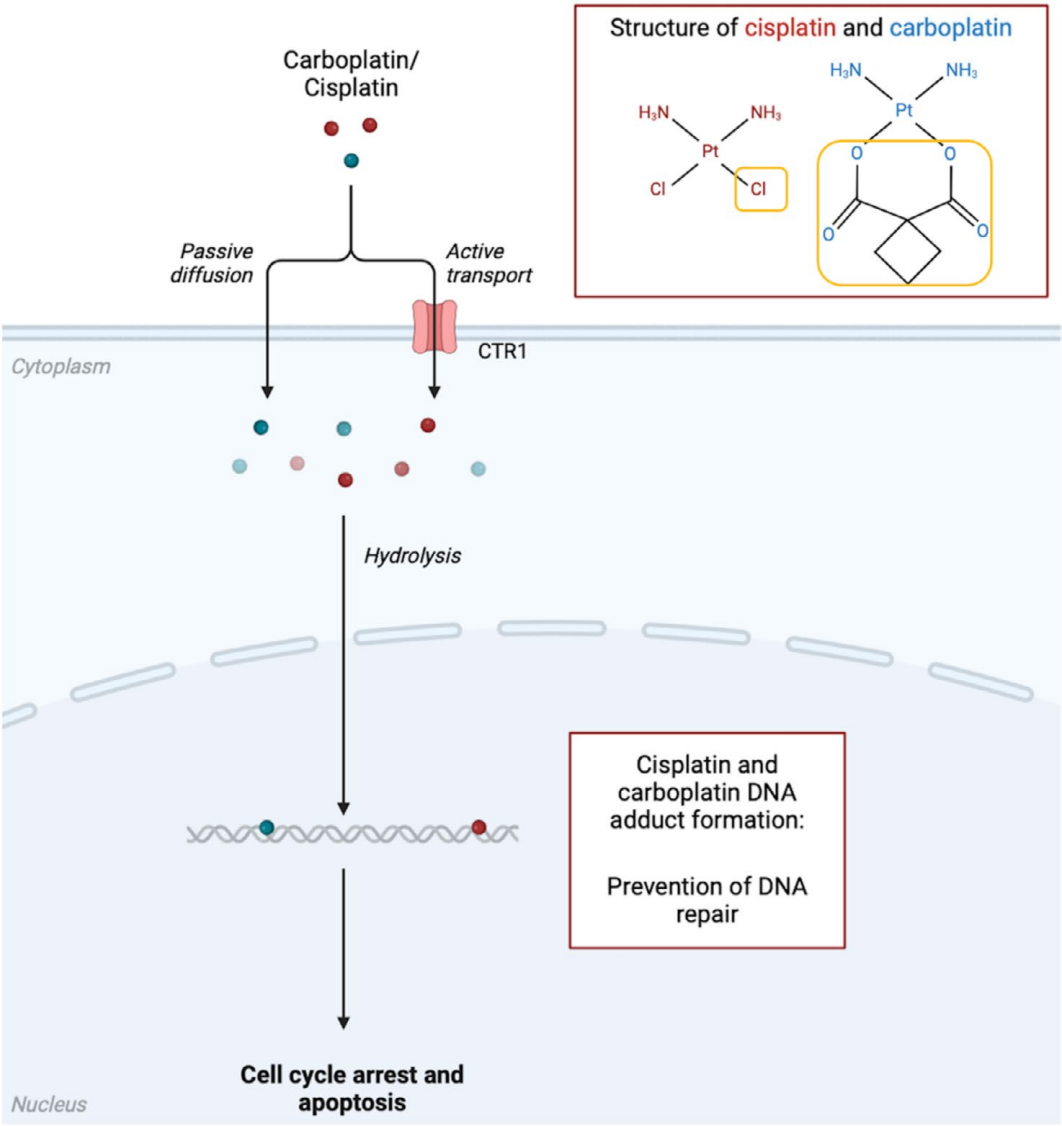
Representation of carboplatin and cisplatin's mechanisms of action. Carboplatin/cisplatin enters the cell via passive diffusion or active transport, commonly via the CTR1 transporter. Once in the cell, both undergo hydrolysis where water molecules replace their respective leaving groups (outlined in yellow), allowing carboplatin/cisplatin to actively bind to DNA. This DNA binding forms platinum‐DNA adducts that promote DNA‐damaging effects that ultimately result in G2/M cell cycle arrest and cancer cell apoptosis. Cl, Chlorine; CTR1, High‐affinity copper uptake protein 1; DNA, Deoxyribonucleic acid; H, Hydrogen; N, Nitrogen; O, Oxygen; Pt, Platinum. Created with BioRender.com.

The anticancer mechanisms of the two platinum drugs differ primarily in their distinct leaving groups. Initially, the platinum drugs enter the cell via passive diffusion or active transport, depending on the cell/tissue type [9]. Once in the cell, carboplatin and cisplatin undergo hydrolysis, where water molecules replace their respective leaving groups. For cisplatin, this leaving group is chloride, whereas for carboplatin, it is a chelate group (cyclobutane‐1,1‐dicarboxylate (CBDCA)) [34]. Following hydrolysis, the platinum drugs actively bind to deoxyribonucleic acid (DNA) via the formation of a bond at the N7 site on guanosine nucleotides as well as a simultaneous bond to a second adjacent guanosine nucleotide. This binding induces the formation of platinum‐DNA adducts that promote DNA‐damaging effects such as the unwinding and bending of the DNA structure [35]. Such adducts enhance cellular radiosensitivity and prevent DNA replication from occurring primarily in the gap phase (G2)/mitotic phase (M) of the cell cycle. As illustrated in Figure 2, these platinum drug effects ultimately result in apoptosis [36, 37].

Although carboplatin is known to possess slower DNA‐binding kinetics and a lowered reactivity potential [38], the beneficial use of carboplatin in the treatment of many types of cancers, including gynecological cancers, is evident [37].

### 4.2 Clinical Efficacy of Carboplatin in Cervical Cancer

Despite its initial use in the treatment of ovarian cancer [38], recent studies support carboplatin's therapeutic potential for cervical cancer, particularly when administered in combination with either radiotherapy, immunotherapy, or paclitaxel. Table 2 summarises the findings from several clinical trials focused on advanced‐stage cervical cancer patients.

**TABLE 2:** A summarized representation of common carboplatin treatment approaches used in advanced‐stage cervical cancer clinical trials.

Trial/study	Stage	Number of patients	Treatment	Outcome	References
Phase III	IB—IVA	500	Cisplatin chemoradiation alone or induction chemotherapy of carboplatin and paclitaxel prior to cisplatin chemoradiation	Induction chemotherapy before chemoradiation = 5‐year progression‐free survival rate of 73% and an overall survival rate of 80% Chemoradiation alone = 5‐year progression‐free survival rate of 64% and an overall survival rate of 72%	[39]
Phase II	Advanced‐stage Recurrent	35	Camrelizumab = first‐line treatment in combination with paclitaxel carboplatin, followed by camrelizumab maintenance thereafter	Objective response rate of 40% and a disease control rate of 92% overall	[40]
Phase II	IVB Recurrent Persistent	34	Paclitaxel, carboplatin, and bevacizumab	Overall survival rate = 88% Median overall survival = 26 months Acceptable toxicity levels	[41]
Prospective study	≥IIB	76 carbo‐platin and 137 cisplatin	Chemoradiation with either cisplatin or carboplatin	Carboplatin = more favoured tolerance profile with reduced renal toxicity	[42]
Retro‐spective study	IIB—IVA	184	Single‐agent carboplatin and cisplatin with radiotherapy	Carboplatin = similar survival benefits to cisplatin	[43]
Phase III	IVB Persistent Recurrent	126	Carboplatin and paclitaxel combination therapy	Carboplatin‐paclitaxel therapy = equally as effective as cisplatin‐paclitaxel therapy	[44]

Carboplatin combination therapy is highly effective in treating advanced‐stage cervical cancer, demonstrating results that support increased survival rates and lowered toxicity. While treatment strategies for advanced cervical cancer are increasingly shifting from traditional chemotherapy to chemo‐immunotherapy, it remains essential to further investigate the role of carboplatin, especially in combination with other platinum‐based agents such as cisplatin and oxaliplatin, given the demonstrated efficacy of platinum compounds in the treatment of cervical cancer. Understanding these interactions could potentially help clarify carboplatin's role within the evolving treatment landscape.

### 4.3 Comparative Analysis of Carboplatin With Other Platinum‐Based Chemotherapy Drugs

**FIGURE 3:**
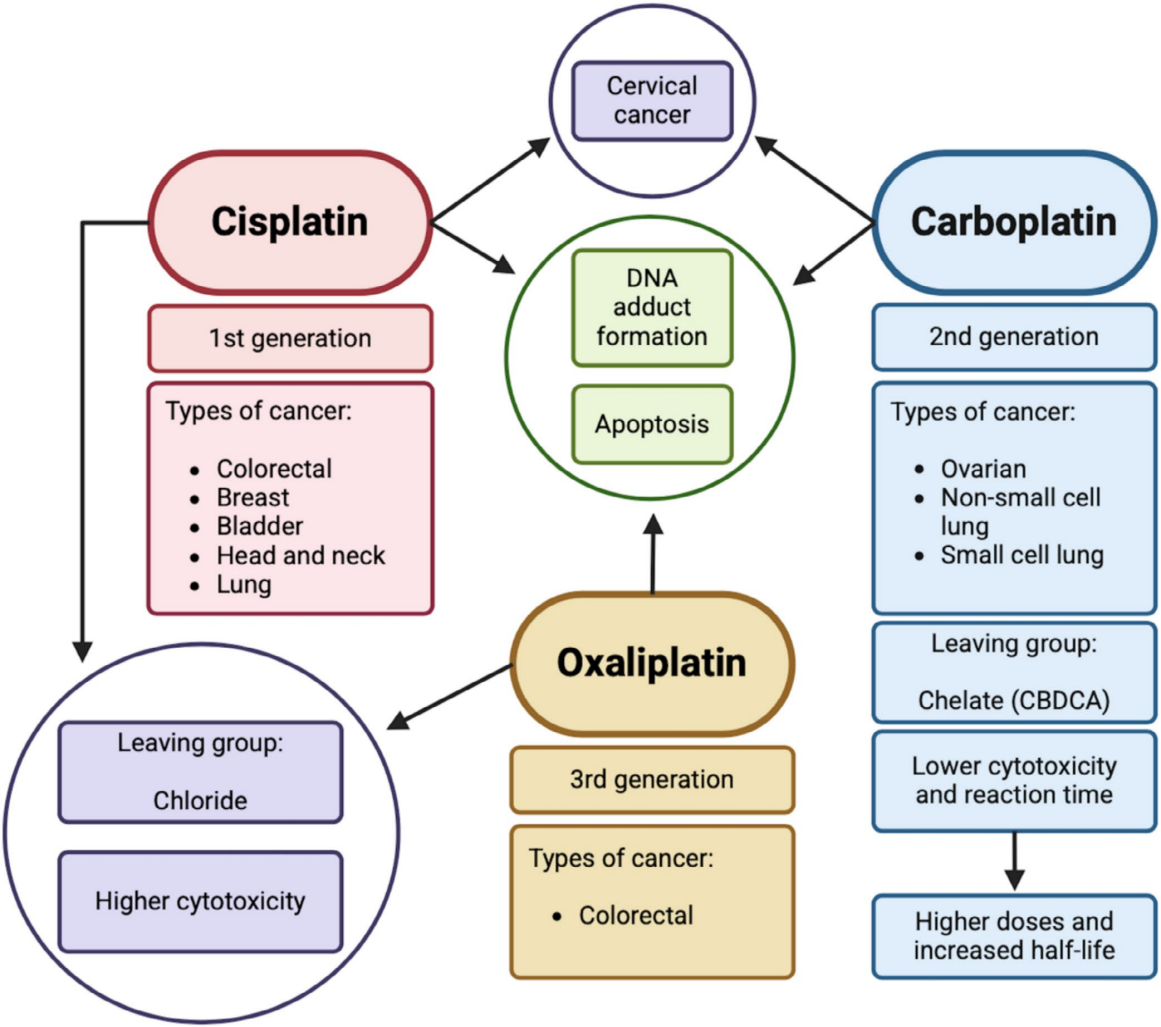
The similarities and differences of the three most commonly prescribed platinum‐based chemotherapeutic drugs. A diagrammatic representation of the shared and distinct characteristics of carboplatin, cisplatin, and oxaliplatin. CBDCA, Cyclobutane‐1,1‐dicarboxylate; DNA, Deoxyribonucleic acid. Created with BioRender.com.

It is observed that 46% of all cancer patients in hospitals receive platinum‐based drug treatment, either alone or in combination with other chemotherapeutic drugs [35]. The discovery of platinum drugs originated in the late 1960s with the clinical introduction of cisplatin, the pioneering agent in this class for cancer treatment [45]. Following this, cisplatin derivatives, namely carboplatin and oxaliplatin, were introduced in hopes of mitigating the observed toxic and mutagenic side effects following cisplatin treatment [46]. Despite all platinum drugs exhibiting anticancer potential through DNA intercalation, crosslinking, and adduct formation [47], each drug exhibits distinct characteristics, as illustrated in Figure 3.

All three platinum drugs preferentially target specific cancer types, exhibiting both distinct preferences and some overlap among them. For instance, cisplatin is commonly used in the treatment of colorectal, ovarian, breast [45], bladder, head and neck, and lung cancer, while carboplatin is commonly used for the treatment of advanced non‐small cell lung cancer, small cell lung cancer, and ovarian cancer [33]. Within recent years, both carboplatin and cisplatin have proven effective in the treatment of both early‐ and advanced‐stage cervical cancer [37]. In contrast, oxaliplatin is not commonly used in cervical cancer treatment but is primarily employed in managing colorectal cancer [47]. Notably, colorectal cancer shares some overlapping molecular and pathological traits with cervical cancer [31], such as CD44v6 expression, which could inform potential cross‐applications of treatment strategies. Cisplatin and oxaliplatin share similarities in their mechanism of action through the loss of a chloride group during hydrolysis in comparison to the loss of the chelating group (CBDCA) of carboplatin [47]. Despite their differences, all three drugs converge on promoting apoptosis. Platinum‐based agents are particularly notable for their ability to induce cell death independently of p53, though they activate a p53‐dependent apoptotic pathway when p53 is functional [33].

Carboplatin is known to possess the lowest degree of toxicity in comparison to cisplatin and oxaliplatin, which have similar cytotoxic effects [33]. However, this reduced toxicity renders the need to administer higher concentrations of carboplatin to achieve the same degree of efficacy [47]. For example, the recommended clinical dose of carboplatin is in a 4:1 ratio with cisplatin [38]. In addition, there is a notable decrease in carboplatin's reactivity time. This is in lieu of the enhanced stability of carboplatin's chelate leaving group in comparison to a chloride leaving group [33].

In addition to DNA crosslinking, platinum drugs are known to form DNA adducts with other cellular structures such as RNA, lipids, and proteins [37]. Knowing this, the reduced reactivity of carboplatin can be seen as advantageous, as it results in a prolonged half‐life of approximately 30 h. This extended duration is due to a lower rate of protein‐carboplatin complex excretion. As a result, carboplatin offers more sustained therapeutic effects compared to cisplatin, which has a half‐life ranging from 1.5 to 3.6 h [38]. Therefore, carboplatin therapy has proven to be highly effective in treating advanced‐stage cervical cancer, demonstrating results that are comparable to, if not more advantageous than, those of cisplatin therapy. Consequently, these findings suggest that carboplatin represents a promising alternative treatment option to cisplatin for patients with cervical cancer.

### 4.4 Limitations of Carboplatin

While carboplatin effectively addresses tumorigenesis with a lower toxicity profile than cisplatin, it is still associated with adverse side effects, including myelosuppression, nephrotoxicity, and neurotoxicity, which may limit its clinical applicability [34]. Furthermore, increased drug resistance and reduced drug sensitivity are common outcomes following repeated exposure to carboplatin therapy [45]. These side effects complicate treatment decisions for cervical cancer patients, particularly given that the recurrence rate for advanced‐stage cases can be as high as 70% [48].

Carboplatin drug resistance can be attributed to cell membrane transporter availability, enhanced DNA repair mechanisms, and enhanced drug efflux [49]. Platinum drug uptake is highly dependent on the cell/tissue type. For example, cell membrane transporters that facilitate platinum drug entry and efflux, such as copper transporter 1 (CTR1) and ATPase copper transporting beta/alpha (ATP7B/A), respectively, are found in varying concentrations on different cell types [9]. ATP7A is predominantly expressed in epithelial cells, while ATP7B is mainly found in brain, liver, or kidney cells. ATP7B is known to promote the efflux of carboplatin out of the cell, thereby aiding in treatment resistance [9]. Thus, the expression level of ATP7B plays a critical role in determining intracellular drug accumulation and, consequently, the effectiveness of platinum‐based chemotherapies. In cancers such as colon and breast, ATP7B is frequently overexpressed [50], contributing to reduced retention of drugs like carboplatin and the development of chemoresistance. In contrast, ATP7B expression appears to be relatively low in cervical cancer [50]. This lower expression may allow for greater intracellular accumulation of carboplatin in cervical cancer cells, potentially enhancing both its cytotoxic effects and therapeutic sensitivity. The known expression of CTR1 on the surface of cervical cancer cells is indicative of the efficacy of platinum drugs. To illustrate this, a study performed by Zisowsky et al. (2007) observed an association between low CTR1 expression levels and diminished intracellular platinum accumulation concentrations, ultimately resulting in reduced cervical cancer cell sensitivity to cisplatin [51]. Furthermore, the presence of CAFs within the TME can prevent the intracellular accumulation of cisplatin and carboplatin due to their CTR1/2 expression regulation potential [36]. Moreover, cellular platinum drug resistance and sensitivity are dependent on the capability of cancer cells to repair platinum drug‐induced DNA damage. For instance, high mRNA expression levels of excision repair cross‐complementation group 1 (ERCC1) can be utilized as a potential biomarker for platinum drug treatment resistance in cervical cancer patients [37].

Platinum drugs are typically administered via infusion to help manage side effects and address potential issues with poor solubility [35]. The recent introduction of platinum drug nanoparticle delivery systems has come to light as a possible method of combating treatment resistance, side effects, and poor solubility. For example, platinum‐based nanocarriers, micelles, and nanostructures/particles are all being investigated as possible modes to achieve optimal carboplatin delivery in cervical cancer patients [52]. Moreover, evidence supporting techniques to counteract carboplatin‐induced myelosuppression is being brought to light, such as the administration of carboplatin in combination with paclitaxel at fractionated doses [37]. In addition, there is a notable absence of studies examining the effects of carboplatin on CD44‐positive cervical cancer cells, which presents important implications for challenges in oncological treatment. Consequently, exploring the mechanisms of action of carboplatin on CD44‐positive cancer cells in other malignancies may reveal promising avenues for future research in cervical cancer.

### 4.5 CD44's Influence on the Effectiveness of Carboplatin in Cancer

Studies highlight the attractive nature of the use of carboplatin as a treatment scheme for advanced‐stage cervical cancer patients (Table 2). However, various types of cancers have shown increased carboplatin resistance in the presence of CD44‐positive cells, while CD44 inhibition has been associated with enhanced carboplatin sensitization.

A study conducted by Elzarkaa et al. (2016) observed increased carboplatin resistance in patients with CD44‐positive advanced‐stage epithelial ovarian cancer compared to CD44‐negative patients [53]. In contrast, a study on prostate cancer by Li et al. (2016) found increased sensitization to carboplatin treatment following CD44 inhibition [54]. Furthermore, they identified CD44 as a glycolytic regulator that resulted in increased ROS, cell proliferation, and glucose metabolism. This finding is particularly concerning, as the rate of glycolysis in cancer cells is often a strong indicator of tumor aggressiveness [54]. In addition to the challenges associated with carboplatin monotherapy in CD44‐positive cancer cells, research highlights the tumorigenic potential of CD44 following chemoradiation therapy with carboplatin. For instance, a study on head and neck squamous cell carcinoma by Baschnagel et al. (2017) found that elevated CD44 levels were linked to poor loco‐regional control and undesirable disease prognosis [55].

Taken together, these findings across multiple cancer types underscore the central role of CD44 in mediating carboplatin resistance and tumor progression. Given that CD44 is similarly overexpressed in cervical cancer, particularly in HPV‐driven cases, these insights provide a compelling rationale for investigating CD44‐targeted strategies to overcome chemoresistance and improve therapeutic outcomes in cervical cancer. In alignment with these findings and considering CD44's potential role in enhancing treatment resistance and metastasis in cervical cancer cells (Figure 1), CD44 shows promise as a predictive biomarker for treatment outcomes in cervical cancer. Another promising approach to reducing the toxic side effects of chemotherapy is through the incorporation of natural products with known anticancer properties. Among these, resveratrol has shown significant potential, and further research is warranted to explore its role as a treatment strategy to overcome CD44‐induced metastasis and treatment resistance.

## 5 Resveratrol: A Potential Therapeutic and Preventive Agent

Cervical cancer treatment regimes, such as the use of chemotherapy, can often be toxic to healthy cells and display a vast array of undesirable patient side effects, including metastasis and cancer recurrence, as mentioned in the previous sections [56]. Therefore, the need to establish treatment options that are less toxic, effective, and accessible is in high demand [57].

### 5.1 Overview of Resveratrol

**FIGURE 4:**
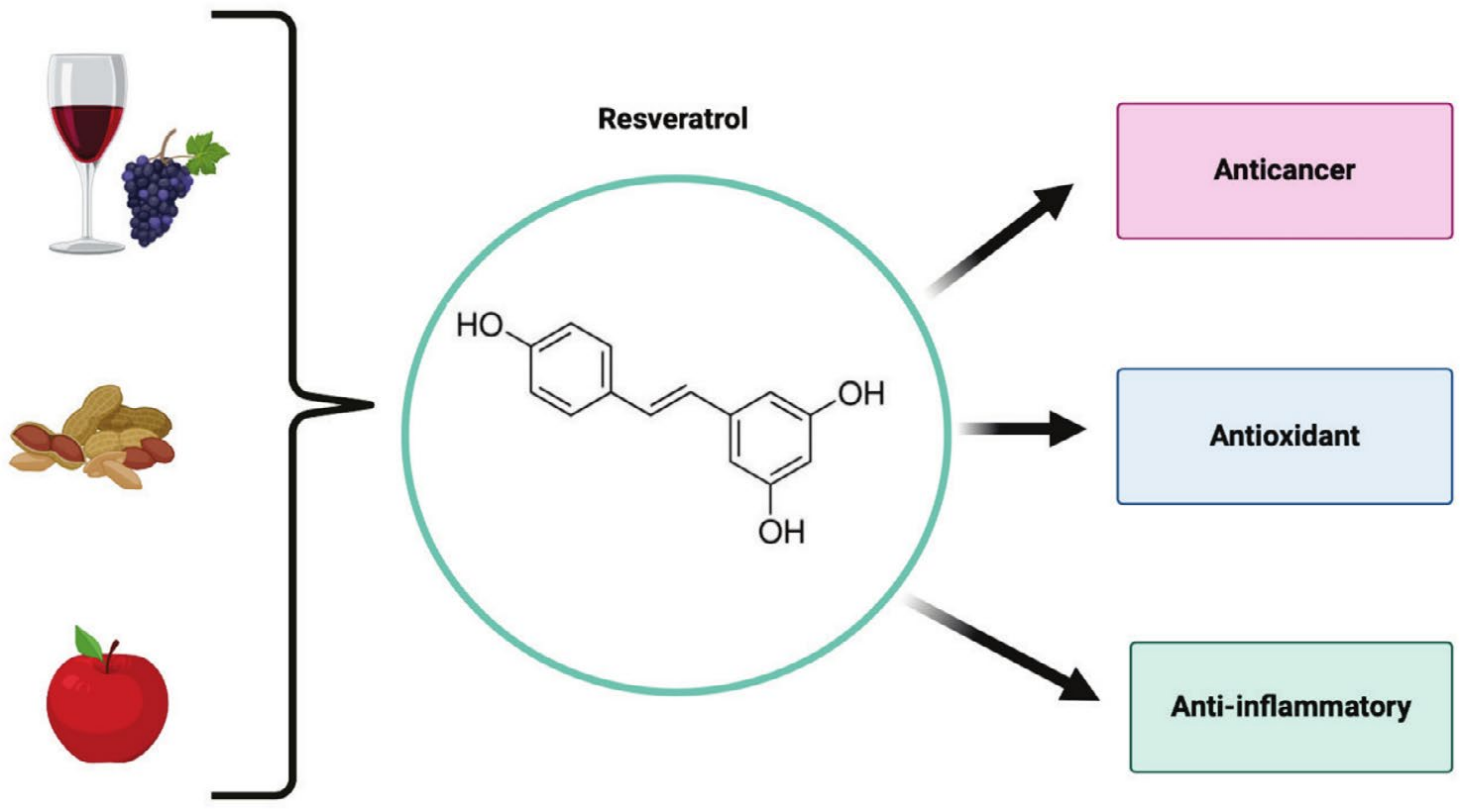
A schematic illustration of dietary sources of resveratrol and its associated health benefits. Created with BioRender.com.

A common low‐toxicity treatment solution, which has been ongoing for centuries, is the use of natural substances [58] as an alternative to or in conjunction with chemotherapeutic drugs. For example, the polyphenolic compound curcumin has demonstrated promising potential in the treatment of cervical cancer [59]. The traditional use of such bioactive compounds has yielded positive therapeutic results via their potential to elicit anticancer, antioxidant, and anti‐inflammatory properties [60]. The naturally occurring polyphenolic stilbene and phytoalexin compound, resveratrol (3,5,4′ – trihydroxy stilbene) (Figure 4), is believed to offer anticancer/chemoprevention mechanisms all while safeguarding the integrity of healthy cells [57, 61].

Resveratrol is typically isolated from the roots of the Japanese knotweed or the white hellebore plant. However, it can be found in abundance in various foods such as grapes, peanuts, berries, red wine, and apples [60]. Due to a wide range of existing resveratrol studies [60], a variety of advantages associated with the use of resveratrol as a natural medicinal remedy are prevalent. So much so that scientific advances have been made, where, in addition to resveratrol's natural abundance, chemical synthesis of resveratrol in its unadulterated form has been accomplished. Thus, providing an ample amount of substance for biological therapeutic use [58]. Furthermore, resveratrol exists as two geometric isomers, with *cis*‐(*Z*) and *trans*‐(*E*) conformation [62]. Interestingly, the *trans*‐conformation can be converted to the *cis*‐conformation upon exposure to heat and ultraviolet radiation [62]. However, *trans*‐resveratrol is more widely understood, with studies highlighting its significant antitumour, antimetastatic, and drug sensitivity‐inducing potential [57, 62]. Although the *trans* isomer is known to possess greater anticancer mechanisms, both isomers have been seen to increase apoptosis within cancer cells through poly(ADP‐ribose) polymerase (PARP) and caspase 3 upregulation [63]. Research surrounding the full anticancer effects of the *cis*‐resveratrol isoform still needs to be fully elucidated. In contrast, extensive research has examined resveratrol's mechanisms of action in cancer treatment, demonstrating its potential benefits, particularly in the management of cervical cancer [56].

### 5.2 Mechanisms of Resveratrol in Cancer Prevention and Treatment

Resveratrol induces its anticancer effects through various mechanisms of action, such as (1) metastasis and EMT pathway inhibition [56, 60, 64], (2) apoptosis induction [65], and (3) its pro‐ and antioxidant potential [66].

#### 5.2.1 The Regulation of Metastasis, Apoptosis, and Epithelial‐To‐Mesenchymal Transition (EMT)

The process of metastasis encapsulates the core underpinnings of drug‐induced treatment resistance within patients. Thus, an existing causal relationship between resveratrol and metastasis could be significant.

Multiple resveratrol studies support decreased metastatic invasion in an array of cancers, namely, head and neck, prostate, gastric, and colorectal, to name a few [61]. Mechanisms promoting the inhibition of cellular proliferation following treatment with resveratrol are commonly observed, such as the inhibition of wingless (Wnt)/β‐catenin, MAPK/extracellular signal‐related kinase (ERK) [56, 60], nuclear factor kappa B (NF‐kB), and transforming growth factor‐β (TGF‐β) signaling pathways [65]. Furthermore, due to metastasis being prompted by the upregulation of mutated growth factor receptor signaling (enhanced epithelial growth factor receptor (EGFR) and vascular endothelial growth factor receptor (VEGFR) expression), resveratrol's inhibition potential could further prevent uncontrolled metastatic cascades [56]. In addition, this resveratrol‐induced prevention causes the inhibition of an important metastatic role player, EMT. A decrease among EMT‐promoting markers, namely, vimentin and N‐cadherin, with simultaneous upregulation of the production of E‐cadherin upon resveratrol treatment has been identified [60]. Moreover, resveratrol inhibits chaotic metastasis‐promoting signaling pathways that feed EMT processes, such as Notch, NF‐kB, Hedgehog, Wnt, phosphoinositide 3‐kinase (PI3K)/protein Kinase B (Akt)/mammalian Target of Rapamycin (mTOR), and TGF‐β pathways [56].

Studies highlighting resveratrol's potential to inhibit metastatic signaling, whilst simultaneously upregulating apoptosis in cervical cancer, are evident [56] (Table 3). In addition, related to cervical cancer specifically, resveratrol decreases both *E6* and *E7* oncogene production, thereby preventing cell proliferation and cell survival signaling pathways, such as the EGFR, NF‐kB, ERK, VEGFR, and signal transducer and activator of transcription 3 (STAT3) pathways [60] as summarized in Table 3.

**TABLE 3:** A tabulated summary of in vitro and in vivo cervical cancer studies with the use of resveratrol as a treatment strategy.

In vitro		
Cell line type and classification	Mechanism of action	References
HeLa – Adenocarcinoma (HPV18) [67]	Increased cell cycle arrest and apoptosis. Increased cytotoxicity. Increased E‐cadherin. Decreased cellular proliferation and cell growth. Decreased levels of oncoprotein E6 and cell migration. Decreased phosphorylated ERK levels. Decreased vimentin, N‐cadherin, metastasis, and wound healing potential. Decreased levels of EGFR. Decreased E2F levels.	[68, 69, 70, 71, 72, 73, 74, 75, 76]
SiHa—Squamous cell carcinoma (HPV16) [67]	Increased E‐cadherin, apoptosis, and cell cycle arrest. Decreased vimentin, N‐cadherin, metastasis, proliferation, wound healing potential, and migration and invasion.	[65, 77]
CaSki—Squamous cell carcinoma (HPV16) [67]	Increased apoptosis, oncoprotein E6 and E7 levels, and cell cycle arrest. Decreased E2F levels and cellular proliferation.	[74]
HT‐3—Cervical carcinoma (HPV negative) [78]	Increased apoptosis. Decreased cellular growth and proliferation.	[65]
W12—Precancerous cervical epithelial cells (HPV16) [79]	Decreased cellular proliferation.	[80]

In vivo		
Model	Mechanism of action	References
Mouse – injected with HeLa cells.	Increased E‐cadherin tumour levels. Increased levels of apoptosis. Decreased vimentin, N‐cadherin, tumour volume, and tumour weight. Decreased tumour levels of oncoprotein E6 and E7.	[70, 72, 74, 81]
Mouse—Injected with HPV oncogene *E6* and *E7* positive cells.	Increased cell cycle arrest. Decreased tumour VEGF levels, tumour size, and tumour oncoprotein E6 levels.	[82]

Abbreviations: EGFR, Epithelial growth factor receptor; ERK, Extracellular signal‐regulated kinase; VEGF, Vascular endothelial growth factor.

Chatterjee et al. (2018) observed decreased cellular proliferation rates, owing to reduced *E6* oncogene levels, following resveratrol treatment in the cervical cancer HeLa cell line [69]. Both Sun et al. (2020) and Shin et al. (2020) found similar results [72, 83]. Inhibition of such pathways suggests resveratrol's ability to inhibit EMT signalling and, therefore, metastasis associated with cervical pathogenesis.

#### 5.2.2 Resveratrol as a Pro‐ and Antioxidant

The dual capability of resveratrol to act as both an antioxidant and a pro‐oxidant is compelling [66]. At low treatment concentrations, such as in our diets, resveratrol is believed to function as an antioxidant, thereby decreasing cellular oxidative stress levels as well as promoting cellular proliferation of cells, specifically non‐malignant cells. In contrast, high treatment concentrations of resveratrol, typically administered in tablet form, have been observed to promote its pro‐oxidant abilities by enhancing mitochondrial reactive oxygen species (ROS) levels and apoptosis [66, 84]. Therefore, resveratrol's pro‐oxidant ability can function to attenuate tumor cell apoptosis via the exploitation of the high levels of ROS present within the TME [85] beyond a critical threshold. In contrast, healthy cells generally possess lower ROS levels [86], rendering them less susceptible to the pro‐oxidant effects of resveratrol. Consequently, high concentrations of resveratrol are unlikely to push the ROS levels in non‐malignant cells beyond the threshold, thereby safeguarding them while selectively targeting cancerous cells.

Thus, resveratrol's mechanisms of action underscore its therapeutic potential against malignant cells. Further exploration of its synergistic and chemo‐sensitizing effects in conjunction with carboplatin is warranted.

### 5.3 The Synergistic and Chemo‐Sensitising Effects of Resveratrol With Carboplatin

Chemo‐sensitisation remains an effective therapeutic approach for overcoming chemoresistance by observing how the use of one drug facilitates the functioning of another [87]. Although no studies currently exist that support the chemo‐sensitising mechanisms of resveratrol for carboplatin therapy in cervical cancer, documented literature on its more toxic counterparts, cisplatin, as well as on oxaliplatin in other cancer types, can be used to infer the potential effects of carboplatin, given that both are platinum‐based drugs.

The use of platinum drugs, such as cisplatin, in combination with natural products has shown beneficial treatment outcomes in many types of cancer [87]. A study exploring the apoptotic effects of combining cisplatin and resveratrol in non‐small cell lung cancer found that combination therapy was more effective than cisplatin monotherapy [87]. The ability of resveratrol to not only enhance the efficacy of cisplatin but also reduce its toxic effects on healthy cells makes this combination therapeutically attractive [88]. Moreover, research supporting resveratrol's ability to enhance chemo‐sensitivity to cisplatin in breast cancer cells and ovarian cancer cells is prevalent [89, 90], where an ovarian cancer study performed by Engelke et al. (2016) explored the cytotoxicity potential of 48‐h resveratrol pre‐treatment, followed by cisplatin therapy [91]. Although there are studies that support synergistic and antagonistic effects of cisplatin and resveratrol co‐treatment, a study performed by Rezk et al. (2006) [92] found the timing of resveratrol administration to be an important factor to consider [93]. This was demonstrated in ovarian cancer, where a comparison between resveratrol and cisplatin co‐treatment and resveratrol pre‐treatment revealed reduced toxicity to healthy cells, along with enhanced chemotherapy‐induced apoptotic effects. Nessa et al. (2012)'s findings coincide with those of Rezk et al. as they observed how pre‐treatment with resveratrol sensitised ovarian cancer cells to platinum‐based chemotherapy treatment, namely, oxaliplatin and cisplatin, through NF‐kB downregulation [93]. In contrast, resveratrol post‐platinum drug treatment has also exhibited the potential to blunt the increased toxicity of oxaliplatin in colon cancer [94]. Clinical trial evidence of resveratrol's effect on cervical cancer has not been investigated. However, clinical trials surrounding the use of resveratrol as a potential treatment option have mostly been conducted on colon cancer and breast cancer and have a common focus on investigating resveratrol's optimal dosage, long‐term effects, and timing for administration [95]. Notably, both preclinical and clinical data support the tumorigenesis‐inhibiting benefits of resveratrol treatment. For example, evidence indicates that resveratrol may downregulate Wnt signaling and cell proliferation in patients with colon pathology (NCT00578396) [96]. In addition, a preclinical study on ovarian cancer by Bjorkland et al. (2011) demonstrated that resveratrol could reduce the required doses of platinum‐based chemotherapy while preserving its apoptotic effects on cancer cells [97].

Another important aspect to consider is the slower reaction rate of carboplatin compared to cisplatin [38]. This delayed action could provide a crucial window for resveratrol to exert its sensitising effects on chemoresistant cell populations, such as CD44‐positive CSCs, or within the tumour microenvironment. This slower onset might allow resveratrol to synergise more effectively with carboplatin, enhancing the therapeutic response and potentially reducing metastasis in cervical cancer. Furthermore, research has shown that resveratrol can modulate p53 activity to improve the apoptotic potential of chemotherapy drugs and increase tumour cell sensitisation [98]. For instance, resveratrol has been shown to regulate p53 expression in several cancers, including lung [99], colorectal [100], and breast cancer [101], where p53 is often inactivated. Its potential to reactivate p53 in these contexts can lead to cell apoptosis and cell cycle arrest [74], which is especially useful in cancers where p53 has been mutated. In the case of cervical cancer, p53 is functionally inactivated by the E6 oncoprotein encoded by high‐risk HPV subtypes [74]. Therefore, resveratrol's demonstrated ability to modulate p53 in other cancer types raises the possibility that it could similarly restore p53 function in HPV‐driven cervical cancer, potentially sensitising cells to chemotherapeutic agents such as carboplatin and cisplatin, thereby enhancing treatment efficacy. Building on this, studies in cervical cancer have demonstrated distinct expression patterns between HPV16 and HPV18 [102, 103]. HPV16 typically shows higher oncoprotein levels of E6/E7 mRNA [102], whereas HPV18 may be associated with more potent p53 degradation due to elevated expression of full‐length E6 protein [103]. These molecular differences may contribute to varying levels of chemoresistance in HPV‐driven cancers. The divergence in E6/E7 oncoprotein expression between HPV16‐ and HPV18‐positive cervical cancers underscores the importance of HPV genotyping in predicting therapeutic response and supports the rationale for using resveratrol as a targeted chemo‐sensitiser in HPV‐associated malignancies.

These findings suggest resveratrol's anticancer effects are contingent upon the type of cancer and the timing of its administration. Furthermore, resveratrol's ability to mitigate the treatment resistance and metastatic effects of CD44 allows for opportunistic chemo‐sensitizing effects of cervical cancer cells to carboplatin.

## 6 The Role of CD44 in Therapeutic Response

Research indicates that cancer cells with high CD44 expression exhibit increased treatment resistance and metastatic potential [24, 25]. This underscores the need to explore how CD44 interactions might influence therapeutic outcomes, particularly concerning resveratrol.

### 6.1 Interactions Between CD44 and Resveratrol

Although studies surrounding resveratrol's anti‐cervical cancer potential exist (Table 3), studies investigating resveratrol's relationship with CD44‐promoting metastasis and chemoresistance mechanisms in cervical cancer remain to be fully elucidated.

Recently, resveratrol has garnered considerable attention for its ability to target CSCs. Although the exact mechanisms of how resveratrol regulates CSC signaling remain unknown, studies investigating this relationship are ongoing [56]. The stemness potential of CSCs is often accompanied by treatment resistance; however, resveratrol has been shown to mitigate chemoresistance in both CSCs and non‐CSCs by increasing their cellular sensitivity to chemotherapy drugs [56]. EI‐Benhawy et al. (2021) demonstrated this through the downregulation of CD44 expression in prostate cancer cells following treatment with resveratrol [104]. Furthermore, Buhrmann et al. (2018) found similar findings with resveratrol treatment in colorectal cancer, leading to decreased CSC abundance [64]. In addition, they observed corresponding decreased EMT activity with reduced vimentin and enhanced E‐cadherin levels. They postulated that this is due to resveratrol's ability to inhibit NF‐kB and TGF‐β signaling pathways. Moreover, Moshiri et al. (2017) came to similar conclusions in mammary gland cells that support resveratrol's ability to inhibit TGF‐β‐induced EMT [105]. Moreover, they found epithelial‐type splicing of CD44 following treatment with resveratrol, thus reducing cellular migration and adhesion effects. In another study, resveratrol decreased stem cell marker *aldehyde dehydrogenase 1* (*ALDH1*) gene activity and CD44 expression in oral CSCs at different concentrations [106]. In support of this, a study conducted in head and neck cancer demonstrated that resveratrol significantly reduced CD44 expression [107]. Notably, both cervical cancer and head and neck squamous cell carcinoma (HNSCC) frequently exhibit upregulation of CD44, largely driven by the oncogenic activity of high‐risk HPV *E6* and *E7* proteins [24, 107]. In addition, resveratrol has been shown to suppress the Wnt/β‐catenin signaling pathway, a fundamental driver of cancer stemness and chemoresistance, in both cancer types [108, 109]. These overlapping molecular features suggest that findings from HNSCC may be translatable to cervical cancer, supporting the rationale for investigating resveratrol's therapeutic potential in this context. Interestingly, a colon cancer study conducted by El‐Readi et al. (2019) demonstrates resveratrol's potential to inhibit the treatment‐resistance effects of *MDR1* [110], thereby promoting drug sensitivity [111] and inhibiting CD44‐acquired drug resistance [25]. Evidence supporting resveratrol's potential to suppress CD44 and inhibit metastasis has gained significant attention in cancer therapy research. However, studies investigating the therapeutic effect of resveratrol when acting upon CD44‐positive cervical tumors are limited, as well as evidence supporting the beneficial use of resveratrol as a sensitizing agent to carboplatin treatment in cervical cancer. Thus, an opportunistic niche exists within the field of cancer research to investigate this effectual relationship to potentially aid as a treatment strategy for advanced‐stage cervical cancer patients. To further refine this approach, it is essential to examine a critical yet often overlooked factor: the role of HPV status in shaping these therapeutic responses.

### 6.2 CD44 and HPV

Emerging evidence suggests that the anticancer effects of resveratrol in cervical cancer may surpass HPV status, yet its efficacy could be modulated by the presence of the CSC marker, CD44 [112].

Interestingly, HPV‐positive cervical cancer cell lines, such as HeLa (HPV18) and CaSki (HPV16), portray high CD44 expression. Whereas the HPV‐negative C33A cell line shows little to no detectable CD44 levels [112]. This differential expression raises the possibility that HPV oncoproteins may influence CD44 regulation, potentially by activating EMT‐associated transcription factors or modulating the tumor microenvironment [29]. Given that CD44 is associated with chemoresistance, metastasis, and stem‐like properties, the therapeutic effects of resveratrol, specifically its ability to target CSC populations and enhance carboplatin sensitivity, may be more pronounced in CD44‐high, HPV‐positive contexts. Therefore, while resveratrol demonstrates HPV‐independent anticancer activity [113], its dual ability to modulate both general tumor biology and CD44‐mediated CSC pathways positions it as a promising option for targeted interventions, especially in HPV‐driven, CD44‐positive cervical cancers. Further investigation into the interplay between HPV status, CD44 expression, and resveratrol responsiveness could provide valuable insight for stratified therapy approaches.

## 7 Discussion: Potential for Chemo‐Sensitization Therapies Targeting CD44 With Carboplatin and Resveratrol

Before gaining an understanding of resveratrol's chemo‐sensitizing potential for cervical cancer cells, it is essential to first predict a possible relationship between CD44 and resveratrol in the context of cervical cancer.

**FIGURE 5:**
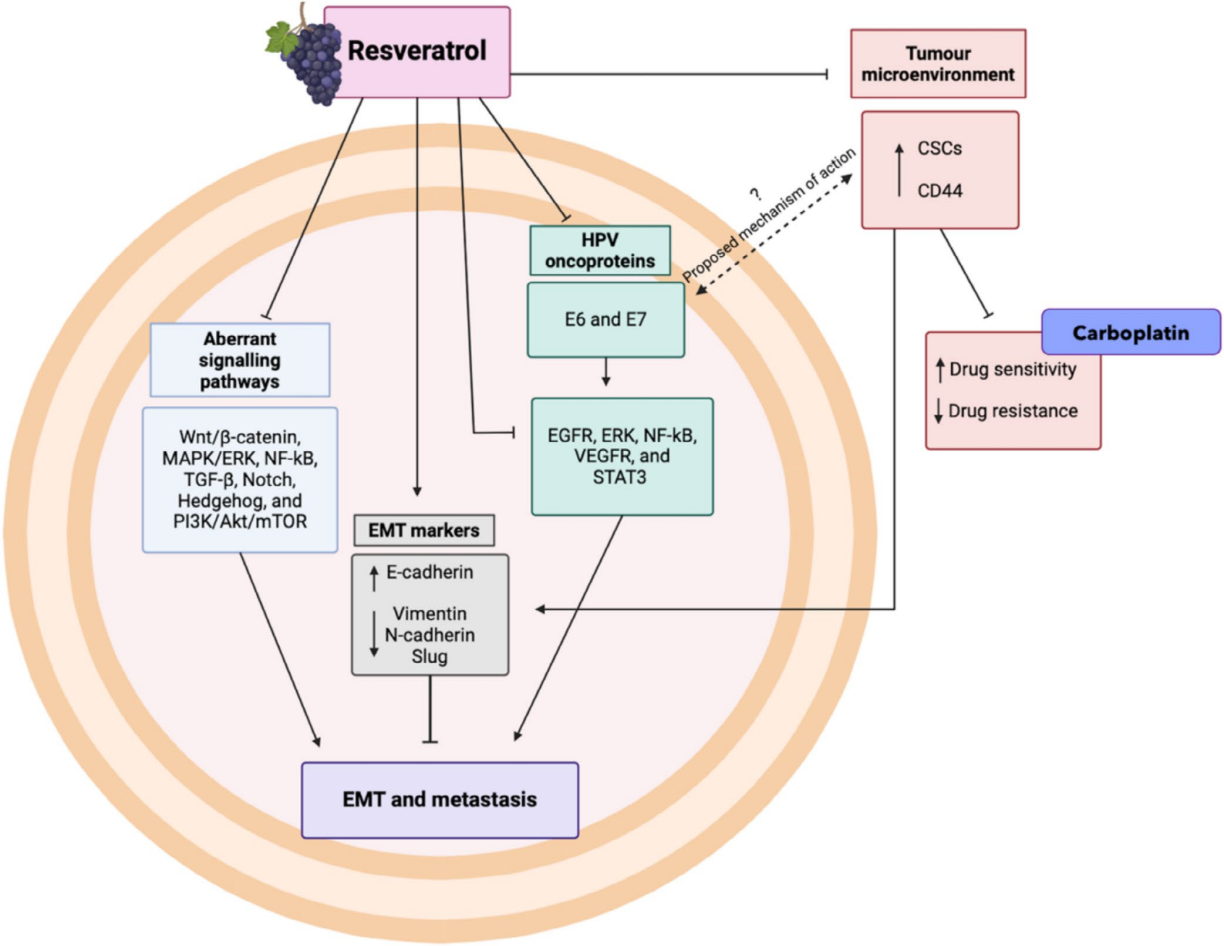
Resveratrol's therapeutic effects on cervical cancer cells. Resveratrol functions to reduce metastasis and EMT processes through aberrant signalling pathway inhibition, epithelial marker E‐cadherin transcription promotion, and reduced mesenchymal marker expression (vimentin, N‐cadherin, and slug) where resveratrol acts directly or via CSC upregulation, inhibition of oncoprotein E6 and E7 production and their associated signalling pathways, and lastly, resveratrol functions to inhibit an increase in the number of CSCs and subsequent CD44 levels found within the tumour microenvironment. The CSC and CD44 inhibition promote increased carboplatin drug sensitivity of cervical cancer cells as well as reduced drug resistance. The proposed mechanism suggests the relationship between resveratrol, CD44, and HPV‐induced cervical pathogenesis drawn from conclusions found within the literature. Akt/PKB, Protein kinase B; CD44, Cluster of differentiation 44; CSC, Cancer stem cell; EGFR, Epidermal growth factor receptor; EMT, Epithelial‐to‐mesenchymal transition; ERK, extracellular signal‐regulated kinase; HPV, Human papillomavirus; MAPK, mitogen‐activated protein kinase; mTOR, mammalian target of rapamycin; NF‐kB, Nuclear factor‐Kappa B; PI3K, phosphatidylinositol 3‐kinase; STAT3, signal transducer and activator of transcription 3; TGF‐β, Transforming growth factor‐beta; VEGFR, Vascular endothelial growth factor receptor; Wnt, Wingless‐related integration site. Created with BioRender.com.

Even though a beneficial treatment relationship between CD44 and resveratrol exists in various other cancer types, no evidence within the field of cervical cancer suggests the role CD44 might play alongside resveratrol's treatment mechanisms. However, deductions based on the individual relationship resveratrol has with HPV, and with CD44‐positive CSCs, could prompt the understanding of a potential CD44‐resveratrol relationship as illustrated in Figure 5, ‘Proposed mechanism of action’.

Resveratrol aids in reducing metastasis and EMT processes within cancer cells as it acts as an aberrant signalling pathway inhibitor [56, 60], promotes the transcription of epithelial marker E‐cadherin [77], and reduces mesenchymal marker expression such as vimentin, N‐cadherin, and slug [72]. Moreover, resveratrol's CSC and CD44 inhibition ability favours increased drug sensitivity of cervical cancer cells as well as reduced drug resistance [56, 57]. In addition, the literature supports an exponential relationship between CSC abundance and CD44 levels within cervical tumours as well as the ability of high‐risk HPV oncogenes *E6* and *E7* to further promote an increase in CSCs [27], and thus a subsequent increase in CD44 expression. However, treatment with resveratrol has been shown to counteract this occurrence via its ability to promote a decrease in *E6* and *E7* oncogene expression levels, as was observed by Sun et al. (2020)'s findings [72]. This could therefore suggest a corresponding reduction in CD44 expression as well as enhanced chemo‐sensitivity within cervical cancer cells. Hence, conclusions drawn are influenced by the literature, suggesting the ‘proposed mechanism of action’ encompasses the effectual relationship between resveratrol, CD44, and HPV‐induced cervical pathogenesis.

Knowing this, it can be inferred that resveratrol possesses the potential to inhibit CD44‐induced metastasis and treatment resistance within cervical cancer cells, whilst indirectly enhancing sensitivity to chemotherapy drugs such as carboplatin. Hence, considering resveratrol's ability to counteract the factors contributing to CD44‐induced treatment resistance (refer to Figure 1), enhance cancer cell apoptosis through increased ROS, and improve the efficacy of platinum‐based chemotherapeutic drugs highlights its potential as a chemo‐sensitizer for carboplatin treatment in advanced or metastatic stage cervical cancer.

## 8 Conclusion

In summary, the abundant natural polyphenolic composition of resveratrol, along with its known anticancer potential, presents a promising therapeutic strategy against the aggressive, heterogeneous nature of metastatic/advanced‐stage cervical cancer. Its potential to inhibit CD44‐induced metastatic/EMT‐induced treatment resistance whilst simultaneously promoting chemo‐sensitizing effects could provide patients with a better quality of life and treatment success rate. Furthermore, this approach potentially aligns with the goals of personalized medicine, aiming to tailor treatments to target specific molecular pathways of individual patient profiles for more effective and targeted cancer therapy. Further clinical research is needed to validate the anticancer effects of resveratrol on CD44 and to assess its potential as a targeted treatment option, in conjunction with carboplatin, for patients with advanced‐stage cervical cancer. This can ultimately result in improved treatment outcomes, quality of life, and prolonged survival for these advanced‐stage cervical cancer patients.

## 9 Challenges and Future Perspectives

Current research on CD44, carboplatin, and resveratrol is limited due to the lack of clinical trial evidence, both long‐term and short‐term, as well as minimal in vitro and in vivo studies specifically examining their combined effects in the treatment of cervical cancer. Furthermore, the precise mechanisms through which carboplatin and resveratrol target treatment resistance and metastasis remain poorly understood. However, CD44 holds significant promise as a potential biomarker for cervical cancer treatment, although its role is influenced by individual patient cancer heterogeneity and the unique conditions of the TME. To move forward, comprehensive research utilizing advanced in vitro and in vivo models, though unable to fully replicate the complexity of the human TME, is crucial for evaluating the potential toxicity and side effects of combining resveratrol with carboplatin. Such studies are a necessary precursor to the design and execution of clinical trials. Addressing these gaps through focused research will not only deepen our understanding of the therapeutic potential of CD44, carboplatin, and resveratrol but also pave the way for more effective, personalized treatments for cervical cancer.

Prospectively, as cervical cancer treatment continues to advance, it is essential to explore complementary therapeutic strategies that address persistent challenges such as chemoresistance and metastasis. This review emphasizes the promising, yet underexplored, potential of targeting CD44 using resveratrol in combination with carboplatin, a strategy that could function synergistically with emerging therapeutic modalities. Notably, immunotherapeutic strategies such as immune checkpoint inhibitors, including pembrolizumab in combination with chemotherapy, are gaining traction as part of the evolving standard of care for recurrent disease [114, 115]. At the same time, the integration of artificial intelligence into early detection and diagnostic workflows is poised to significantly enhance accuracy and accessibility. For instance, the CerviXpert model, a convolutional neural network capable of classifying cervical abnormalities with remarkable precision (~98%), represents a scalable solution particularly suited for resource‐limited settings [116]. As highlighted in recent pharmacotherapeutic reviews, future treatment paradigms are shifting toward a more integrated approach, combining targeted agents, immunotherapy, and digital tools to personalize cervical cancer care [114]. Together, these advancements underscore a future where cervical cancer management is more personalized, precise, accessible, and anchored not only in novel therapeutic agents but also in cutting‐edge technologies that bridge gaps in global health equity.

## Author Contributions

Article idea: Cayleigh de Sousa; Conceptualisation: Carla Eksteen; Investigating: Cayleigh de Sousa; Writing – original draft preparation: Cayleigh de Sousa; Writing – review and editing: Anna‐Mart Engelbrecht, Carla Eksteen; Funding acquisition: Anna‐Mart Engelbrecht; Supervision: Anna‐Mart Engelbrecht; Visualisation: Cayleigh de Sousa; Project administration: Anna‐Mart Engelbrecht.

## Ethics Statement

The authors have nothing to report.

## Consent

The authors have nothing to report.

## Conflicts of Interest

The authors declare no conflicts of interest.

## Data Availability

The authors have nothing to report.

## References

[1] D. Singh , J. Vignat , V. Lorenzoni , et al., “Global Estimates of Incidence and Mortality of Cervical Cancer in 2020: A Baseline Analysis of the WHO Global Cervical Cancer Elimination Initiative,” Lancet Global Health 11, no. 2 (2023): e197–e206. 36528031 10.1016/S2214-109X(22)00501-0 PMC9848409

[2] R. Hull , M. Mbele , T. Makhafola , et al., “Cervical Cancer in Low and Middleincome Countries,” Oncology Letters 20, no. 3 (2020): 2058–2074. 32782524 10.3892/ol.2020.11754 PMC7400218

[3] M. A. Oyervides‐Muñoz , A. A. Pérez‐Maya , H. F. Rodríguez‐Gutiérrez , et al., “Understanding the HPV Integration and Its Progression to Cervical Cancer,” Infection, Genetics and Evolution 61 (2018): 134–144. 10.1016/j.meegid.2018.03.003 29518579

[4] S. Zhang , H. Xu , L. Zhang , and Y. Qiao , “Cervical Cancer: Epidemiology, Risk Factors and Screening,” Chinese Journal of Cancer Research 32, no. 6 (2020): 720–728. 33446995 10.21147/j.issn.1000-9604.2020.06.05 PMC7797226

[5] P. A. Cohen , A. Jhingran , A. Oaknin , and L. Denny , “Cervical Cancer,” Lancet 393, no. 10167 (2019): 169–182. 30638582 10.1016/S0140-6736(18)32470-X

[6] I. A. George , R. Chauhan , R. E. Dhawale , et al., “Insights Into Therapy Resistance in Cervical Cancer,” Advances in Cancer Biology‐Metastasis 6 (2022): 100074.

[7] L. Kumar , P. Harish , P. S. Malik , and S. Khurana , “Chemotherapy and Targeted Therapy in the Management of Cervical Cancer,” Current Problems in Cancer 42, no. 2 (2018): 120–128. 29530393 10.1016/j.currproblcancer.2018.01.016

[8] A. Chao , C. T. Lin , and C. H. Lai , “Updates in Systemic Treatment for Metastatic Cervical Cancer,” Current Treatment Options in Oncology 15 (2014): 1–13. 24429797 10.1007/s11864-013-0273-1

[9] G. F. D. Sousa , S. R. Wlodarczyk , and G. Monteiro , “Carboplatin: Molecular Mechanisms of Action Associated With Chemoresistance,” Brazilian Journal of Pharmaceutical Sciences 50, no. 4 (2014): 693–701.

[10] J. Fares , M. Y. Fares , H. H. Khachfe , H. A. Salhab , and Y. Fares , “Molecular Principles of Metastasis: A Hallmark of Cancer Revisited,” Signal Transduction and Targeted Therapy 5, no. 1 (2020): 28. 32296047 10.1038/s41392-020-0134-x PMC7067809

[11] S. Gupta , P. Kumar , and B. C. Das , “HPV: Molecular Pathways and Targets,” Current Problems in Cancer 42, no. 2 (2018): 161–174. 29706467 10.1016/j.currproblcancer.2018.03.003

[12] J. Majidpoor and K. Mortezaee , “Steps in Metastasis: An Updated Review,” Medical Oncology 38 (2021): 1–17. 10.1007/s12032-020-01447-w 33394200

[13] E. L. Ivanova , B. Costa , T. Eisemann , et al., “CD44 Expressed by Myeloid Cells Promotes Glioma Invasion,” Frontiers in Oncology 12 (2022): 969787. 35992852 10.3389/fonc.2022.969787 PMC9386454

[14] C. Chen , S. Zhao , A. Karnad , and J. W. Freeman , “The Biology and Role of CD44 in Cancer Progression: Therapeutic Implications,” Journal of Hematology & Oncology 11 (2018): 1–23. 29747682 10.1186/s13045-018-0605-5 PMC5946470

[15] M. Hassn Mesrati , S. E. Syafruddin , M. A. Mohtar , and A. Syahir , “CD44: A Multifunctional Mediator of Cancer Progression,” Biomolecules 11, no. 12 (2021): 1850. 34944493 10.3390/biom11121850 PMC8699317

[16] H. K. Mehdi , K. Raju , and S. R. Sheela , “Association of P16, Ki‐67, and CD44 Expression in High‐Grade Squamous Intraepithelial Neoplasia and Squamous Cell Carcinoma of the Cervix,” Journal of Cancer Research and Therapeutics 19, no. Suppl 1 (2023): S260–S267. 37148002 10.4103/jcrt.jcrt_43_21

[17] M. N. Fahmi , I. N. Hertapanndika , and F. Kusuma , “The Prognostic Value of Cancer Stem Cell Markers in Cervical Cancer: A Systematic Review and Meta‐Analysis,” Asian Pacific Journal of Cancer Prevention: APJCP 22, no. 12 (2021): 4057–4065. 34967589 10.31557/APJCP.2021.22.12.4057 PMC9080387

[18] S. Chopra , K. Deodhar , V. Pai , et al., “Cancer Stem Cells, CD44, and Outcomes Following Chemoradiation in Locally Advanced Cervical Cancer: Results From a Prospective Study,” International Journal of Radiation Oncology, Biology, Physics 103, no. 1 (2019): 161–168. 30213750 10.1016/j.ijrobp.2018.09.003

[19] J. Leone , J. E. Perez , M. E. Dominguez , et al., “Role of Difucosylated Lewis Y Antigen in Outcome of Locally Advanced Cervical Squamous Cell Carcinoma Treated With Cisplatin Regimen,” International Journal of Biological Markers 31, no. 3 (2016): 300–308. 10.5301/jbm.5000206 27197582

[20] S. Dasari , W. Rajendra , and L. Valluru , “Evaluation of Soluble CD44 Protein Marker to Distinguish the Premalignant and Malignant Carcinoma Cases in Cervical Cancer Patients,” Medical Oncology 31 (2014): 1–7. 10.1007/s12032-014-0139-9 25064733

[21] S. Costa , P. Terzano , A. Bovicelli , et al., “CD44 Isoform 6 (CD44v6) is a Prognostic Indicator of the Response to Neoadjuvant Chemotherapy in Cervical Carcinoma,” Gynecologic Oncology 80, no. 1 (2001): 67–73. 11136572 10.1006/gyno.2000.6016

[22] L. T. Senbanjo and M. A. Chellaiah , “CD44: A Multifunctional Cell Surface Adhesion Receptor Is a Regulator of Progression and Metastasis of Cancer Cells,” Frontiers in Cell and Developmental Biology 5 (2017): 18. 28326306 10.3389/fcell.2017.00018 PMC5339222

[23] H. Xu , M. Niu , X. Yuan , K. Wu , and A. Liu , “CD44 as a Tumor Biomarker and Therapeutic Target,” Experimental Hematology & Oncology 9, no. 1 (2020): 1–14. 33303029 10.1186/s40164-020-00192-0 PMC7727191

[24] N. Sudhalkar , N. P. Rathod , A. Mathews , et al., “Potential Role of Cancer Stem Cells as Biomarkers and Therapeutic Targets in Cervical Cancer,” Cancer Reports 2, no. 2 (2019): e1144. 32721115 10.1002/cnr2.1144 PMC7941515

[25] Z. Yaghobi , A. Movassaghpour , M. Talebi , et al., “The Role of CD44 in Cancer Chemoresistance: A Concise Review,” European Journal of Pharmacology 903 (2021): 174147. 33961871 10.1016/j.ejphar.2021.174147

[26] Z. Wu , J. Lu , A. Loo , et al., “Role of CD44 in Chemotherapy Treatment Outcome: A Scoping Review of Clinical Studies,” International Journal of Molecular Sciences 25, no. 6 (2024): 3141. 38542115 10.3390/ijms25063141 PMC10970610

[27] J. OrganistaNava , Y. GómezGómez , O. L. GaribayCerdenares , M. A. LeyvaVázquez , and B. IlladesAguiar , “Cervical Cancer Stem Cell‐Associated Genes: Prognostic Implications in Cervical Cancer,” Oncology Letters 18, no. 1 (2019): 7–14. 31289465 10.3892/ol.2019.10307 PMC6540231

[28] J. X. Lin , C. Yoon , P. Li , et al., “Increased CD44 Expression and MEK Activity Predict Worse Prognosis in Gastric Adenocarcinoma Patients Undergoing Gastrectomy,” Journal of Gastrointestinal Surgery 25, no. 5 (2021): 1147–1155. 32410176 10.1007/s11605-020-04616-4

[29] A. Martincuks , P. C. Li , Q. Zhao , et al., “CD44 in Ovarian Cancer Progression and Therapy Resistance—A Critical Role for STAT3,” Frontiers in Oncology 10 (2020): 589601. 33335857 10.3389/fonc.2020.589601 PMC7736609

[30] P. Dall , A. Hekele , H. Ikenberg , et al., “Increasing Incidence of CD44v7/8 Epitope Expression During Uterine Cervical Carcinogenesis,” International Journal of Cancer 69, no. 2 (1996): 79–85. 8608987 10.1002/(SICI)1097-0215(19960422)69:2<79::AID-IJC2>3.0.CO;2-S

[31] L. Ma , L. Dong , and P. Chang , “CD44v6 Engages in Colorectal Cancer Progression,” Cell Death & Disease 10, no. 1 (2019): 30. 30631039 10.1038/s41419-018-1265-7 PMC6328617

[32] J. Paken , C. D. Govender , M. Pillay , M. Feyasa , and V. Sewram , “Cisplatin‐Associated Ototoxicity Amongst Cervical Cancer Patients: A Prospective Cohort Study in South Africa,” PLoS One 18, no. 4 (2023): e0283639. 37014872 10.1371/journal.pone.0283639 PMC10072443

[33] S. Schoch , S. Gajewski , J. Rothfuß , A. Hartwig , and B. Köberle , “Comparative Study of the Mode of Action of Clinically Approved Platinum‐Based Chemotherapeutics,” International Journal of Molecular Sciences 21, no. 18 (2020): 6928. 32967255 10.3390/ijms21186928 PMC7555145

[34] P. Štarha , J. Vančo , and Z. Trávníček , “Platinum Iodido Complexes: A Comprehensive Overview of Anticancer Activity and Mechanisms of Action,” Coordination Chemistry Reviews 380 (2019): 103–135.

[35] S. Alassadi , M. J. Pisani , and N. J. Wheate , “A Chemical Perspective on the Clinical Use of Platinum‐Based Anticancer Drugs,” Dalton Transactions 51, no. 29 (2022): 10835–10846. 35781551 10.1039/d2dt01875f

[36] J. Mikuła‐Pietrasik , A. Witucka , M. Pakuła , et al., “Comprehensive Review on How Platinum‐ and Taxane‐Based Chemotherapy of Ovarian Cancer Affects Biology of Normal Cells,” Cellular and Molecular Life Sciences 76 (2019): 681–697. 30382284 10.1007/s00018-018-2954-1 PMC6514066

[37] G. Y. Ho , N. Woodward , and J. I. Coward , “Cisplatin Versus Carboplatin: Comparative Review of Therapeutic Management in Solid Malignancies,” Critical Reviews in Oncology/Hematology 102 (2016): 37–46. 27105947 10.1016/j.critrevonc.2016.03.014

[38] S. Dasari and P. B. Tchounwou , “Cisplatin in Cancer Therapy: Molecular Mechanisms of Action,” European Journal of Pharmacology 740 (2014): 364–378. 25058905 10.1016/j.ejphar.2014.07.025 PMC4146684

[39] M. McCormack , D. G. Rincón , G. Eminowicz , et al., “LBA8 A Randomised Phase III Trial of Induction Chemotherapy Followed by Chemoradiation Compared With Chemoradiation Alone in Locally Advanced Cervical Cancer: The GCIG INTERLACE Trial,” Annals of Oncology 34 (2023): S1276.

[40] X. Zhang , J. Chen , N. Liu , et al., “Camrelizumab (SHR‐1210) With Carboplatin and Albumin‐Binding Paclitaxel in Patients With Metastatic or Recurrent Cervical Cancer: An Open‐Label, Phase 2 Trial,” Journal of Cancer Research and Therapeutics 18, no. 2 (2022): 482–487. 35645118 10.4103/jcrt.jcrt_1851_21

[41] K. Suzuki , S. Nagao , T. Shibutani , et al., “Phase II Trial of Paclitaxel, Carboplatin, and Bevacizumab for Advanced or Recurrent Cervical Cancer,” Gynecologic Oncology 154, no. 3 (2019): 554–557. 31285082 10.1016/j.ygyno.2019.05.018

[42] E. Tharavichitkul , V. Lorvidhaya , P. Kamnerdsupaphon , et al., “Combined Chemoradiation of Cisplatin Versus Carboplatin in Cervical Carcinoma: A Single Institution Experience From Thailand,” BMC Cancer 16 (2016): 1–9. 10.1186/s12885-016-2558-9 PMC4950639 27435245

[43] A. M. Sebastião , L. S. da Silva Rocha , R. D. Gimenez , et al., “Carboplatin‐Based Chemoradiotherapy in Advanced Cervical Cancer: An Alternative to Cisplatin‐Based Regimen?,” European Journal of Obstetrics & Gynecology and Reproductive Biology 201 (2016): 161–165. 27137353 10.1016/j.ejogrb.2016.03.016

[44] R. Kitagawa , N. Katsumata , T. Shibata , et al., “A Randomized, Phase III Trial of Paclitaxel Plus Carboplatin (TC) Versus Paclitaxel Plus Cisplatin (TP) in Stage IVb, Persistent or Recurrent Cervical Cancer: Japan Clinical Oncology Group Study (JCOG0505),” (2012). 10.1093/jjco/hyp117 19825815

[45] C. Zhang , C. Xu , X. Gao , and Q. Yao , “Platinum‐Based Drugs for Cancer Therapy and Anti‐Tumor Strategies,” Theranostics 12, no. 5 (2022): 2115–2132. 35265202 10.7150/thno.69424 PMC8899578

[46] B. Szikriszt , Á. Póti , E. Németh , N. Kanu , C. Swanton , and D. Szüts , “Cisplatin Is More Mutagenic Than Carboplatin or Oxaliplatin at Equitoxic Concentrations. bioRxiv,” (2020), pp.2020‐08.

[47] B. Szefler and P. Czeleń , “Will the Interactions of Some Platinum (II)‐Based Drugs With B‐Vitamins Reduce Their Therapeutic Effect in Cancer Patients? Comparison of Chemotherapeutic Agents Such as Cisplatin, Carboplatin and Oxaliplatin—A Review,” International Journal of Molecular Sciences 24, no. 2 (2023): 1548. 36675064 10.3390/ijms24021548 PMC9862491

[48] X. Chao , X. Song , H. Wu , Y. You , M. Wu , and L. Li , “Selection of Treatment Regimens for Recurrent Cervical Cancer,” Frontiers in Oncology 11 (2021): 618485. 33604304 10.3389/fonc.2021.618485 PMC7884815

[49] T. Makovec , “Cisplatin and Beyond: Molecular Mechanisms of Action and Drug Resistance Development in Cancer Chemotherapy,” Radiology and Oncology 53, no. 2 (2019): 148–158. 30956230 10.2478/raon-2019-0018 PMC6572495

[50] Z. Zhang , A. Zhang , Y. Shi , Z. Zhao , and Z. Zhao , “An Association Between ATP7B Expression and Human Cancer Prognosis and Immunotherapy: A Pan‐Cancer Perspective,” BMC Medical Genomics 16, no. 1 (2023): 307. 38037104 10.1186/s12920-023-01714-5 PMC10687837

[51] J. Zisowsky , S. Koegel , S. Leyers , et al., “Relevance of Drug Uptake and Efflux for Cisplatin Sensitivity of Tumor Cells,” Biochemical Pharmacology 73, no. 2 (2007): 298–307. 17097621 10.1016/j.bcp.2006.10.003

[52] M. Pourmadadi , M. M. Eshaghi , M. Shaghaghi , et al., “Nano‐Scale Drug Delivery Systems for Carboplatin: A Comprehensive Review,” OpenNano 13 (2023): 100175.

[53] A. A. Elzarkaa , B. E. Sabaa , D. Abdelkhalik , et al., “Clinical Relevance of CD44 Surface Expression in Advanced Stage Serous Epithelial Ovarian Cancer: A Prospective Study,” Journal of Cancer Research and Clinical Oncology 142 (2016): 949–958. 26762850 10.1007/s00432-016-2116-5 PMC11819065

[54] W. Li , A. Cohen , Y. Sun , et al., “The Role of CD44 in Glucose Metabolism in Prostatic Small Cell Neuroendocrine Carcinoma,” Molecular Cancer Research 14, no. 4 (2016): 344–353. 26832214 10.1158/1541-7786.MCR-15-0466 PMC4834240

[55] A. M. Baschnagel , N. Tonlaar , M. Eskandari , et al., “Combined CD 44, c‐MET, and EGFR Expression in p16‐Positive and p16‐Negative Head and Neck Squamous Cell Carcinomas,” Journal of Oral Pathology & Medicine 46, no. 3 (2017): 208–213. 27442811 10.1111/jop.12478

[56] V. K. Bhaskara , B. Mittal , V. V. Mysorekar , N. Amaresh , and J. Simal‐Gandara , “Resveratrol, Cancer and Cancer Stem Cells: A Review on Past to Future,” Current Research in Food Science 3 (2020): 284–295. 33305295 10.1016/j.crfs.2020.10.004 PMC7718213

[57] J. H. Ko , G. Sethi , J. Y. Um , et al., “The Role of Resveratrol in Cancer Therapy,” International Journal of Molecular Sciences 18, no. 12 (2017): 2589. 29194365 10.3390/ijms18122589 PMC5751192

[58] M. Annaji , I. Poudel , S. H. Boddu , R. D. Arnold , A. K. Tiwari , and R. J. Babu , “Resveratrol‐Loaded Nanomedicines for Cancer Applications,” Cancer Reports 4, no. 3 (2021): e1353. 33655717 10.1002/cnr2.1353 PMC8222557

[59] X. Zhang , L. Zhu , X. Wang , H. Zhang , L. Wang , and L. Xia , “Basic Research on Curcumin in Cervical Cancer: Progress and Perspectives,” Biomedicine & Pharmacotherapy 162 (2023): 114590. 36965256 10.1016/j.biopha.2023.114590

[60] M. Nadile , M. I. Retsidou , K. Gioti , A. Beloukas , and E. Tsiani , “Resveratrol Against Cervical Cancer: Evidence From In Vitro and In Vivo Studies,” Nutrients 14, no. 24 (2022): 5273. 36558430 10.3390/nu14245273 PMC9787601

[61] A. Rauf , M. Imran , M. S. Butt , M. Nadeem , D. G. Peters , and M. S. Mubarak , “Resveratrol as an Anti‐Cancer Agent: A Review,” Critical Reviews in Food Science and Nutrition 58, no. 9 (2018): 1428–1447. 28001084 10.1080/10408398.2016.1263597

[62] C. Leischner , M. Burkard , A. Michel , et al., “Comparative Analysis of the Antitumor Activity of Cis‐ and Trans‐Resveratrol in Human Cancer Cells With Different p53 Status,” Molecules 26, no. 18 (2021): 5586. 34577057 10.3390/molecules26185586 PMC8466563

[63] D. Jeon , M. Jo , Y. Lee , et al., “Inhibition of ANO1 by Cis‐ and Trans‐Resveratrol and Their Anticancer Activity in Human Prostate Cancer PC‐3 Cells,” International Journal of Molecular Sciences 24, no. 2 (2023): 1186. 36674697 10.3390/ijms24021186 PMC9862168

[64] C. Buhrmann , M. Yazdi , B. Popper , et al., “Resveratrol Chemosensitizes TNF‐β‐Induced Survival of 5‐FU‐Treated Colorectal Cancer Cells,” Nutrients 10, no. 7 (2018): 888. 30002278 10.3390/nu10070888 PMC6073304

[65] H. Alharbi , A. S. Alshehri , M. Ahmad , and W. W. Guo , “Promising Anti‐Cervical Carcinoma and Inflammatory Agent, Resveratrol Targets Poly (ADP‐Ribose) Polymerase 1 (PARP‐1) Induced Premature Ovarian Failure With a Potent Enzymatic Modulatory Activity,” Journal of Reproductive Immunology 144 (2021): 103272. 33465522 10.1016/j.jri.2021.103272

[66] M. Ashrafizadeh , S. Taeb , H. Haghi‐Aminjan , et al., “Resveratrol as an Enhancer of Apoptosis in Cancer: A Mechanistic Review,” Anti‐Cancer Agents in Medicinal Chemistry 21, no. 17 (2021): 2327–2336. 33081687 10.2174/1871520620666201020160348

[67] K. Sak , “Characteristic Features of Cytotoxic Activity of Flavonoids on Human Cervical Cancer Cells,” Asian Pacific Journal of Cancer Prevention 15, no. 19 (2014): 8007–8018. 25338977 10.7314/apjcp.2014.15.19.8007

[68] A. Flores‐Pérez and G. Elizondo , “Apoptosis Induction and Inhibition of HeLa Cell Proliferation by Alpha‐Naphthoflavone and Resveratrol Are Aryl Hydrocarbon Receptor‐Independent,” Chemico‐Biological Interactions 281 (2018): 98–105. 29274324 10.1016/j.cbi.2017.12.029

[69] K. Chatterjee , D. AlSharif , C. Mazza , P. Syar , M. Al Sharif , and J. E. Fata , “Resveratrol and Pterostilbene Exhibit Anticancer Properties Involving the Downregulation of HPV Oncoprotein E6 in Cervical Cancer Cells,” Nutrients 10, no. 2 (2018): 243. 29485619 10.3390/nu10020243 PMC5852819

[70] Y. Zhao , X. Yuan , X. Li , and Y. Zhang , “Resveratrol Significantly Inhibits the Occurrence and Development of Cervical Cancer by Regulating Phospholipid Scramblase 1,” Journal of Cellular Biochemistry 120, no. 2 (2019): 1527–1531. 30350320 10.1002/jcb.27335

[71] Z. Liu , Y. Li , G. She , et al., “Resveratrol Induces Cervical Cancer HeLa Cell Apoptosis Through the Activation and Nuclear Translocation Promotion of FOXO3a,” Die Pharmazie—An International Journal of Pharmaceutical Sciences 75, no. 6 (2020): 250–254. 10.1691/ph.2020.0386 32539920

[72] X. Sun , Q. Xu , L. Zeng , et al., “Resveratrol Suppresses the Growth and Metastatic Potential of Cervical Cancer by Inhibiting STAT3Tyr705 Phosphorylation,” Cancer Medicine 9, no. 22 (2020): 8685–8700. 33040485 10.1002/cam4.3510 PMC7666735

[73] S. Pani , A. Sahoo , A. Patra , and P. R. Debata , “Phytocompounds Curcumin, Quercetin, Indole‐3‐Carbinol, and Resveratrol Modulate Lactate–Pyruvate Level Along With Cytotoxic Activity in HeLa Cervical Cancer Cells,” Biotechnology and Applied Biochemistry 68, no. 6 (2021): 1396–1402. 33099806 10.1002/bab.2061

[74] X. Sun , P. Fu , L. Xie , et al., “Resveratrol Inhibits the Progression of Cervical Cancer by Suppressing the Transcription and Expression of HPV E6 and E7 Genes,” International Journal of Molecular Medicine 47, no. 1 (2021): 335–345. 33236130 10.3892/ijmm.2020.4789 PMC7723400

[75] S. Pani , S. Mohapatra , A. Sahoo , B. Baral , and P. R. Debata , “Shifting of Cell Cycle Arrest From the S‐Phase to G2/M Phase and Downregulation of EGFR Expression by Phytochemical Combinations in HeLa Cervical Cancer Cells,” Journal of Biochemical and Molecular Toxicology 36, no. 1 (2022): e22947. 34726804 10.1002/jbt.22947

[76] L. Li , R. L. Qiu , Y. Lin , et al., “Resveratrol Suppresses Human Cervical Carcinoma Cell Proliferation and Elevates Apoptosis via the Mitochondrial and p53 Signaling Pathways,” Oncology Letters 15, no. 6 (2018): 9845–9851. 29928358 10.3892/ol.2018.8571 PMC6004645

[77] H. Nakamura , A. Taguchi , K. Kawana , et al., “Therapeutic Significance of Targeting Survivin in Cervical Cancer and Possibility of Combination Therapy With TRAIL,” Oncotarget 9, no. 17 (2018): 13451–13461. 29568369 10.18632/oncotarget.24413 PMC5862590

[78] F. Yin , N. Wang , S. Wang , et al., “HPV16 Oncogenes E6 or/and E7 May Influence the Methylation Status of RASSFIA Gene Promoter Region in Cervical Cancer Cell Line HT‐3,” Oncology Reports 37, no. 4 (2017): 2324–2334. 28260046 10.3892/or.2017.5465

[79] L. S. Einbond , J. Zhou , K. Huang , et al., “Plant Compounds Inhibit the Growth of W12 Cervical Precancer Cells Containing Episomal or Integrant HPV DNA; Tanshinone IIA Synergizes With Curcumin in Cervical Cancer Cells,” Viruses 17, no. 1 (2024): 55. 39861845 10.3390/v17010055 PMC11768664

[80] L. S. Einbond , J. Zhou , H. A. Wu , et al., “A Novel Cancer Preventative Botanical Mixture, TriCurin, Inhibits Viral Transcripts and the Growth of W12 Cervical Cells Harbouring Extrachromosomal or Integrated HPV16 DNA,” British Journal of Cancer 124, no. 5 (2021): 901–913. 33257842 10.1038/s41416-020-01170-3 PMC7921087

[81] X. Hao , X. Sun , H. Zhu , et al., “Hydroxypropyl‐β‐Cyclodextrin‐Complexed Resveratrol Enhanced Antitumor Activity in a Cervical Cancer Model: In Vivo Analysis,” Frontiers in Pharmacology 12 (2021): 573909. 33935691 10.3389/fphar.2021.573909 PMC8082405

[82] K. Chatterjee , S. Mukherjee , J. Vanmanen , P. Banerjee , and J. E. Fata , “Dietary Polyphenols, Resveratrol and Pterostilbene Exhibit Antitumor Activity on an HPV E6‐Positive Cervical Cancer Model: An In Vitro and In Vivo Analysis,” Frontiers in Oncology 9 (2019): 352. 31143704 10.3389/fonc.2019.00352 PMC6521745

[83] H. J. Shin , J. M. Han , Y. S. Choi , and H. J. Jung , “Pterostilbene Suppresses Both Cancer Cells and Cancer Stem‐Like Cells in Cervical Cancer With Superior Bioavailability to Resveratrol,” Molecules 25, no. 1 (2020): 228. 31935877 10.3390/molecules25010228 PMC6982958

[84] A. Shaito , A. M. Posadino , N. Younes , et al., “Potential Adverse Effects of Resveratrol: A Literature Review,” International Journal of Molecular Sciences 21, no. 6 (2020): 2084. 32197410 10.3390/ijms21062084 PMC7139620

[85] B. M. Sahoo , B. K. Banik , P. Borah , and A. Jain , “Reactive Oxygen Species (ROS): Key Components in Cancer Therapies,” Anti‐Cancer Agents in Medicinal Chemistry 22, no. 2 (2022): 215–222. 34102991 10.2174/1871520621666210608095512

[86] B. Perillo , M. Di Donato , A. Pezone , et al., “ROS in Cancer Therapy: The Bright Side of the Moon,” Experimental & Molecular Medicine 52, no. 2 (2020): 192–203. 32060354 10.1038/s12276-020-0384-2 PMC7062874

[87] V. Cocetta , V. Quagliariello , F. Fiorica , M. Berretta , and M. Montopoli , “Resveratrol as Chemosensitizer Agent: State of Art and Future Perspectives,” International Journal of Molecular Sciences 22, no. 4 (2021): 2049. 33669559 10.3390/ijms22042049 PMC7922064

[88] S. Mirzaei , M. H. Gholami , A. Zabolian , et al., “Resveratrol Augments Doxorubicin and Cisplatin Chemotherapy: A Novel Therapeutic Strategy,” Current Molecular Pharmacology 16, no. 3 (2023): 280–306. 35430977 10.2174/1874467215666220415131344

[89] I. LeonGalicia , J. DiazChavez , M. E. AlbinoSanchez , et al., “Resveratrol Decreases Rad51 Expression and Sensitizes Cisplatinresistant MCF7 Breast Cancer Cells,” Oncology Reports 39, no. 6 (2018): 3025–3033. 29620223 10.3892/or.2018.6336

[90] L. Y. Li Yan , “Effect of Resveratrol on Cisplatin Chemotherapy Sensitivity of Ovarian Cancer Cells,” Journal of International Oncology 45 (2018): 5–9.

[91] L. H. Engelke , A. Hamacher , P. Proksch , and M. U. Kassack , “Ellagic Acid and Resveratrol Prevent the Development of Cisplatin Resistance in the Epithelial Ovarian Cancer Cell Line A2780,” Journal of Cancer 7, no. 4 (2016): 353–363. 26918049 10.7150/jca.13754 PMC4749356

[92] Y. A. Rezk , S. S. Balulad , R. S. Keller , and J. A. Bennett , “Use of Resveratrol to Improve the Effectiveness of Cisplatin and Doxorubicin: Study in Human Gynecologic Cancer Cell Lines and in Rodent Heart,” American Journal of Obstetrics and Gynecology 194, no. 5 (2006): e23–e26. 16647892 10.1016/j.ajog.2005.11.030

[93] M. U. Nessa , P. Beale , C. Chan , J. Q. Yu , and F. Huq , “Combinations of Resveratrol, Cisplatin and Oxaliplatin Applied to Human Ovarian Cancer Cells,” Anticancer Research 32, no. 1 (2012): 53–59. 22213288

[94] D. G. Park , “Antichemosensitizing Effect of Resveratrol in Cotreatment With Oxaliplatin in HCT116 Colon Cancer Cell,” Annals of Surgical Treatment and Research 86, no. 2 (2014): 68–75. 24761411 10.4174/astr.2014.86.2.68 PMC3994604

[95] B. Song , W. Wang , X. Tang , et al., “Inhibitory Potential of Resveratrol in Cancer Metastasis: From Biology to Therapy,” Cancers 15, no. 10 (2023): 2758. 37345095 10.3390/cancers15102758 PMC10216034

[96] R. F. Holcombe , M. Martinez , K. Planutis , and M. Planutiene , “Effects of a Grape‐Supplemented Diet on Proliferation and Wnt Signaling in the Colonic Mucosa Are Greatest for Those Over Age 50 and With High Arginine Consumption,” Nutrition Journal 14 (2015): 1–8. 26085034 10.1186/s12937-015-0050-z PMC4472174

[97] M. Björklund , J. Roos , V. Gogvadze , and M. Shoshan , “Resveratrol Induces SIRT1‐ and Energy–Stress‐Independent Inhibition of Tumor Cell Regrowth After Low‐Dose Platinum Treatment,” Cancer Chemotherapy and Pharmacology 68 (2011): 1459–1467. 21479886 10.1007/s00280-011-1640-x

[98] S. C. Gupta , R. Kannappan , S. Reuter , J. H. Kim , and B. B. Aggarwal , “Chemosensitization of Tumors by Resveratrol,” Annals of the New York Academy of Sciences 1215, no. 1 (2011): 150–160. 21261654 10.1111/j.1749-6632.2010.05852.x PMC3060406

[99] M. Rasheduzzaman , J. K. Jeong , and S. Y. Park , “Resveratrol Sensitizes Lung Cancer Cell to TRAIL by p53 Independent and Suppression of Akt/NF‐κB Signaling,” Life Sciences 208 (2018): 208–220. 30031063 10.1016/j.lfs.2018.07.035

[100] Z. Liu , X. Wu , J. Lv , H. Sun , and F. Zhou , “Resveratrol Induces p53 in Colorectal Cancer Through SET7/9,” Oncology Letters 17, no. 4 (2019): 3783–3789. 30881498 10.3892/ol.2019.10034 PMC6403518

[101] B. Chatterjee , K. Ghosh , and S. R. Kanade , “Resveratrol Modulates Epigenetic Regulators of Promoter Histone Methylation and Acetylation That Restores BRCA1, p53, p21CIP1 in Human Breast Cancer Cell Lines,” BioFactors 45, no. 5 (2019): 818–829. 31317586 10.1002/biof.1544

[102] P. Giorgi Rossi , S. Bisanzi , E. Allia , et al., “Determinants of Viral Oncogene E6‐E7 mRNA Overexpression in a Population‐Based Large Sample of Women Infected by High‐Risk Human Papillomavirus Types,” Journal of Clinical Microbiology 55, no. 4 (2017): 1056–1065. 28100595 10.1128/JCM.01794-16 PMC5377832

[103] S. Baba , A. Taguchi , A. Kawata , et al., “Differential Expression of Human Papillomavirus 16‐, 18‐, 52‐, and 58‐Derived Transcripts in Cervical Intraepithelial Neoplasia,” Virology Journal 17 (2020): 1–10. 32143682 10.1186/s12985-020-01306-0 PMC7060624

[104] S. A. El‐Benhawy , M. I. Morsi , E. I. Fahmy , M. A. Soula , F. F. H. Al Zahraa , and A. R. Arab , “Role of Resveratrol as Radiosensitizer by Targeting Cancer Stem Cells in Radioresistant Prostate Cancer Cells (PC‐3),” Asian Pacific Journal of Cancer Prevention: APJCP 22, no. 12 (2021): 3823. 34967561 10.31557/APJCP.2021.22.12.3823 PMC9080384

[105] A. Moshiri , M. Puppo , M. Rossi , R. Gherzi , and P. Briata , “Resveratrol Limits Epithelial to Mesenchymal Transition Through Modulation of KHSRP/hnRNPA1‐Dependent Alternative Splicing in Mammary Gland Cells,” Biochimica et Biophysica Acta 1860, no. 3 (2017): 291–298. 28088441 10.1016/j.bbagrm.2017.01.001

[106] M. K. Alam , N. R. Alqhtani , B. Alnufaiy , et al., “A Systematic Review and Meta‐Analysis of the Impact of Resveratrol on Oral Cancer: Potential Therapeutic Implications,” BMC Oral Health 24, no. 1 (2024): 412. 38575921 10.1186/s12903-024-04045-8 PMC10993553

[107] T. C. Almeida , G. N. da Silva , D. V. de Souza , et al., “Resveratrol Effects in Oral Cancer Cells: A Comprehensive Review,” Medical Oncology 38 (2021): 1–10. 10.1007/s12032-021-01548-0 34273003

[108] J. Ren , L. Xie , X. Zhu , et al., “Resveratrol Interrupts Wnt/β‐Catenin Signalling in Cervical Cancer by Activating Ten‐Eleven Translocation 5‐Methylcytosine Dioxygenase 1,” Cytokine 190 (2025): 156930. 40188654 10.1016/j.cyto.2025.156930

[109] R. M. Castilho and J. S. Gutkind , “The Wnt/β‐Catenin Signaling Circuitry in Head and Neck Cancer,” in Molecular Determinants of Head and Neck Cancer (Springer New York, 2014), 199–214.

[110] M. Z. El‐Readi , S. Eid , A. A. Abdelghany , H. S. Al‐Amoudi , T. Efferth , and M. Wink , “Resveratrol Mediated Cancer Cell Apoptosis, and Modulation of Multidrug Resistance Proteins and Metabolic Enzymes,” Phytomedicine 55 (2019): 269–281. 30668439 10.1016/j.phymed.2018.06.046

[111] W. H. Talib , A. R. Alsayed , M. Barakat , M. I. Abu‐Taha , and A. I. Mahmod , “Targeting Drug Chemo‐Resistance in Cancer Using Natural Products,” Biomedicine 9 (2021): 1353. 10.3390/biomedicines9101353 PMC8533186 34680470

[112] A. Gutiérrez‐Hoya , O. Zerecero‐Carreón , A. Valle‐Mendiola , et al., “Cervical Cancer Cells Express Markers Associated With Immunosurveillance,” Journal of Immunology Research 2019, no. 1 (2019): 1242979. 31198791 10.1155/2019/1242979 PMC6526527

[113] S. P. García‐Zepeda , E. García‐Villa , J. Díaz‐Chávez , R. Hernández‐Pando , and P. Gariglio , “Resveratrol Induces Cell Death in Cervical Cancer Cells Through Apoptosis and Autophagy,” European Journal of Cancer Prevention 22, no. 6 (2013): 577–584. 23603746 10.1097/CEJ.0b013e328360345f

[114] O. D'Oria , G. Bogani , I. Cuccu , et al., “Pharmacotherapy for the Treatment of Recurrent Cervical Cancer: An Update of the Literature,” Expert Opinion on Pharmacotherapy 25, no. 1 (2024): 55–65. 38159033 10.1080/14656566.2023.2298329

[115] X. Zhang , W. J. Yin , A. L. Zhang , et al., “Meta‐Analysis of Efficacy and Safety of Pembrolizumab for the Treatment of Advanced or Recurrent Cervical Cancer,” Journal of Obstetrics and Gynaecology 44, no. 1 (2024): 2390564. 39150330 10.1080/01443615.2024.2390564

[116] R. S. Akash , R. Islam , S. S. I. Badhon , and K. T. Hossain , “CerviXpert: A Multi‐Structural Convolutional Neural Network for Predicting Cervix Type and Cervical Cell Abnormalities,” DIGITAL HEALTH 10 (2024): 20552076241295440. 39529914 10.1177/20552076241295440 PMC11552049

